# Polythioesters Prepared by Ring‐Opening Polymerization of Cyclic Thioesters and Related Monomers

**DOI:** 10.1002/asia.202200641

**Published:** 2022-07-27

**Authors:** Hui Li, Sophie M. Guillaume, Jean‐François Carpentier

**Affiliations:** ^1^ Univ Rennes CNRS ISCR-UMR 6226 35000 Rennes France

**Keywords:** Thioester, Thiolactone, Ring-Opening Polymerization (ROP), Ring-Opening Copolymerization (ROCOP), Polythioester, Degradability, Recyclability

## Abstract

Polyhydroxyalkanoates (PHAs) are biodegradable and biocompatible polyesters with a wide range of applications; in particular, they currently stand as promising alternatives to conventional polyolefin‐based “plastics”. The introduction of sulfur atoms within the PHAs backbone can endow the resulting polythioesters (PTEs) with differentiated, sometimes enhanced thermal, optical and mechanical properties, thereby widening their versatility and use. Hence, PTEs have been gaining increasing attention over the past half‐decade. This review highlights recent advances towards the synthesis of well‐defined PTEs by ring‐opening polymerization (ROP) of cyclic thioesters – namely thiolactones – as well as of *S*‐carboxyanhydrides and thionolactones; it also covers the ring‐opening copolymerization (ROCOP) of cyclic thioanhydrides or thiolactones with epoxides or episulfides. Most of the ROP reactions described are of anionic type, mediated by inorganic, organic or organometallic initiators/catalysts, along with a few enzymatic reactions as well. Emphasis is placed on the reactivity of the thio monomers, in relation to their ring‐size ranging from 4‐ to 5‐, 6‐ and 7‐membered cycles, the nature of the catalyst/initiating systems implemented and their efficiency in terms of activity and control over the PTE molar mass, dispersity, topology, and microstructure.

## Graphical abstract

**The synthesis of polythioesters** by anionic type ring‐opening polymerization (ROP) of cyclic thioesters, *S*‐carboxyanhydrides, thionolactones as well as by ring‐opening copolymerization (ROCOP) of cyclic thioanhydrides or thiolactones with epoxides or episulfides is described and discussed in relation to the ring‐size of the cyclic monomers.

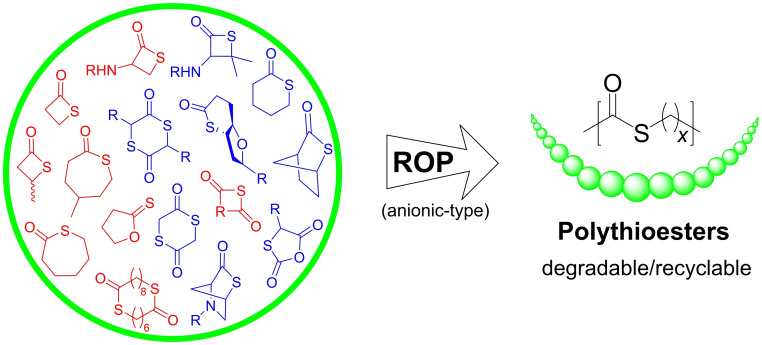

## 1 Introduction

(Bio)degradable polymers, especially polyesters, have received growing attention over the past decades as alternatives to conventional materials such as polyolefins.^[1]^ Among the biodegradable polymers that have been much developed, polyhydroxyalkanoates (PHAs) are of particular and increasing interest. These polymer materials can combine the film‐barrier properties of polyesters with the mechanical performances of fossil resources‐based polyolefins such as polyethylene and polypropylene, and they find a variety of packaging, agricultural, biomedical and pharmaceutical applications due to their biocompatibility and (bio)degradability.^[2]^ However, the development of society generates additional requirements in terms of properties, feeding the need for improved polymer materials.

In this regard, the introduction of sulfur atoms into the polymer backbone can impart polymers with special properties, significantly differentiated from those of regular hydrocarbon‐based polymer analogues.^[3]^ Sulfur‐containing polymers are attractive materials that have recently gained increasing attention because of their unique properties, such as high optical features, metal coordination ability and affinity for metal surfaces, self‐healing capability, and so forth.^[3]^ Among all sulfur‐containing polymers, polythioesters (PTEs) (featuring the (C=O)−S linkage) exhibit valuable properties different from those of their oxoester analogues (*i. e*., polyesters with (C=O)−O linkage), including high refractive index and improved crystallinity.^[3]^ Another much significant characteristic of PTEs is the weak (C=O)−S bond and its intrinsic reactivity; hence, PTEs, in comparison to their corresponding polyester analogues, possess greater potential as dynamic and responsive materials, degradable materials, and even chemically recyclable materials.[^3a^, ^4^]

Aliphatic PTEs can be prepared from the polycondensation of thiocarboxylic acids (−C(=O)SH) by removal of water under azeotropic distillation conditions.^[5]^ Yet, ring‐opening polymerization (ROP) of thiolactones (−C(=O)−S−) is commonly favored because it enables the formation of higher molar mass PTEs under milder reaction conditions. Also, ROP of thiolactones, a chain growth process, allows the preparation of linear aliphatic PTEs better controlled in terms of molecular parameters, such as molar mass, dispersity, and stereoregularity, than the aforementioned step‐growth process.

The present review mainly focuses on recent developments on the ROP and ring‐opening copolymerization (ROCOP) of cyclic thio monomers towards PTEs. It discusses of and is structured according to the variety of PTEs as a function of monomer, namely thiolactones, cyclic thioanhydrides, *S*‐carboxyanhydrides and thionolactones, in relation to their ring size ranging from 4‐ to 5‐, 6‐ and 7‐membered cycles. The vast majority of the ROP reactions described in this review are of anionic type, mediated by inorganic, organic or organometallic initiators/catalysts, along with a few enzymatic reactions as well; it does not cover cationic nor radical type polymerizations of thio monomers, although, when relevant, leading references and/or reviews on these fields are provided.

## 2 Ring‐opening polymerization of 4‐membered β‐propiothiolactones and higher derivatives

### 2.1 Ring‐opening polymerization of β‐propiothiolactone

**Scheme 1:**
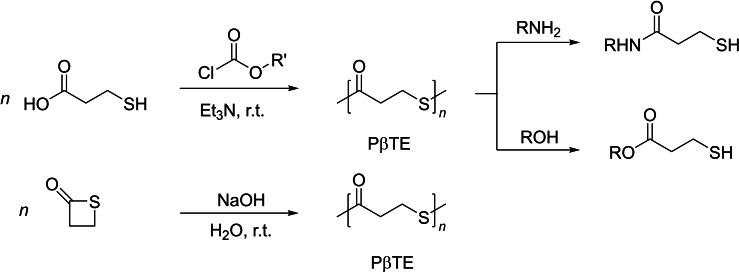
Chemical synthesis of poly(β‐propiothiolactone) (PβTE) from β‐propiothiolactone.^[6]^

Poly(β‐propiothiolactone), the in‐chain sulfur analogue of poly(3‐hydroxypropionate), is often observed as the by‐product or even the main compound incidental to the synthesis of β‐propiothiolactone. For example, the group of Knunyants reported that poly(β‐thioester) (PβTE) formed exclusively from the reaction of β‐thiohydroxy‐propionic acid with a chloroformate in the presence of triethylamine, even in dilute aqueous solution (Scheme 1, top).^[6]^ The chemical structure of the recovered polymer was supported by the formation of the corresponding amides and esters of β‐thiohydroxy‐propionic acid upon its reaction with either amines or alcohols, respectively. β‐Propiothiolactone is also obtained from the reaction of 3‐chloropropionyl chloride with hydrogen sulfide in the presence of an excess of triethylamine in methylene chloride, yet, alongside poly(β‐propiothiolactone) as the major product.^[6]^

β‐Propiothiolactone is briefly reported to “readily polymerize in the presence of small amounts of NaOH in dilute aqueous solution”,^[6]^ giving rise to the corresponding PβTE (Scheme 1, bottom). Yet, the topology (linear or cyclic) and the nature of the terminal groups of the resulting PβTE remain undocumented. The returned PβTE can be easily cleaved by amines giving the corresponding amides of β‐thiohydroxy‐propionic acid, thereby providing a chemical degradation pathway. To our knowledge, the thermal properties of chemically synthesized PβTEs have never been documented. Microbial PβTE, as produced from *E. Coli*. cultured with thioalkanoic acids, melts at 170 °C, a temperature much higher (*ca*.+93 °C) than that of the *O*‐analogous polymer, namely poly(3‐hydroxypropionate).^[7]^

### 2.2 Ring‐opening polymerization of 4‐sulfonamido‐β‐propiothiolactones

**Scheme 2:**
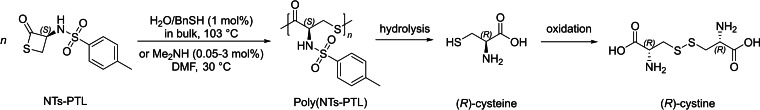
ROP of NTs‐PTL towards optically active PTE.^[8a]^

To date, very few results have been published concerning the preparation and properties of PTEs derived from β‐propiothiolactone derivatives. Early examples tracing back to the 1960s reported the ROP of (*S*)‐α‐*p*‐toluenesulfonamido‐β‐propiothiolactone (NTs‐PTL) either in bulk (i. e., under neat conditions) at a temperature slightly above its melting point, initiated by small amounts (1 mol %) of water or benzyl thioalcohol, or in DMF in the presence of various nucleophilic initiators such as dimethylamine, or even thermally (100 °C) without any initiator, to produce the corresponding optically active poly(NTs‐PTL) (Scheme 2).^[8a]^ However, these polythioesters showed low molar mass values (typically 2,600–12,700 g.mol^−1^), as determined from iodometric titration in DMF solution. This analytical technique did not enable to gain further information on terminal groups fidelity or dispersity.

**Scheme 3:**
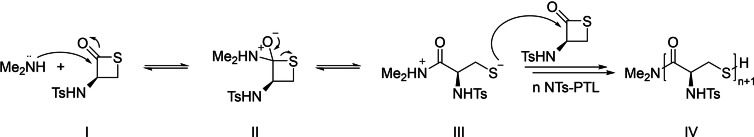
Proposed mechanism for the Me_2_NH‐initiated ROP of NTs‐PTL.^[8a]^

The authors proposed a mechanism (Scheme 3) involving the nucleophilic attack (e. g., amine) onto the electrophilic carbonyl carbon atom of NTs‐PTL monomer (II), thereby opening the ring to form a zwitterionic thiolate‐type intermediate (III); the latter is a strong nucleophile that can further react with additional monomer upon propagation, eventually giving linear PTE. Hydrolysis of the resulting poly(NTs‐PTL) led to (*R*)‐cysteine which was subsequently oxidized to (*R*)‐cystine of the same optical/enantiomeric purity, indicating that such polymerization conditions do not induce racemization of the monomer. It further demonstrates that this class of PβTE can readily degrade into small molecules from which the initial NTs‐PTL monomer can be resynthesized. In light of the pendant reactive amino group, these poly(NTs‐PTL) raised great interest to introduce different functional groups along the PTE backbone, upon chemical modification. Also, non‐amide‐bonded polycysteines containing thioester groups in the main chain are known to play an important role in acylation reactions in biological systems as well.^[9]^

**Scheme 4:**
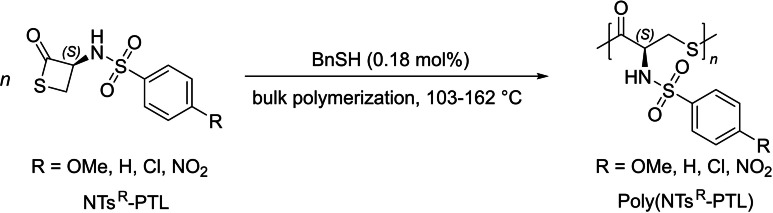
ROP of various α‐acylamino‐β‐thiolactones NTs^R^‐PTLs.^[8b]^

Further studies conducted by the same group evidenced the great impact of the NTs functional groups on their rate of polymerization. A series of α‐(*para‐*substituted‐benzenesulfonamido)‐β‐propiothiolactones, NTs^R^‐PTL, were subjected to ROP initiated by benzyl mercaptan, thereby investigating the electronic contribution of the R *para* substituent (Scheme 4).^[8b]^ Kinetic analysis, by measuring the change of specific rotation as a function of time, indicated that the ROP at room temperature is first order in monomer with *k*_OMe_=2.555, *k*_Me_=2.22, *k*_H_=2.028; *k_Cl_* =1.611, and *k*_NO2_=0.533 mL.g^−1^.h^−1^. It was then proposed that the proton on the nitrogen atom, which acidity is modulated by the *para* substituent, plays a significant role.

To gain further insights into the contribution of this proton to the rate of polymerization through hydrogen bonding, the polymerizability of a set of *N,N*‐disubstituted β‐thiolactones was compared with that of *N*‐monosubstituted analogues.^[8c]^ The rates of conversion, as determined by monitoring the change of specific rotation with time, indicated that the *N*‐tosylamino derivative polymerizes much faster than *N*‐methyl‐*N*‐tosylamino‐β‐thiolactone. The other examples further illustrated the higher polymerizability of *N*‐monosubstituted *vs. N,N*‐disubstituted β‐thiolactones and showed that the rate of the initiating and propagating steps of the ROP of *para*‐substituted benzenesulfonamido‐β‐thiolactones are strongly improved by hydrogen bonding.

**Scheme 5:**
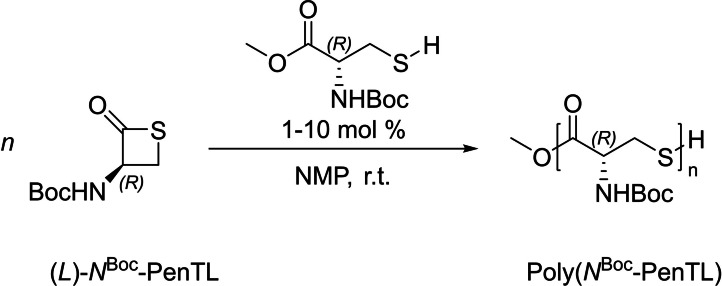
ROP of a *N*‐Boc‐cysteine derived β‐thiolactone (*N* ^Boc^‐PenTL).^[10]^

More recently, the group of Matsuoka insightfully revisited the ROP of a *N*‐Boc‐cysteine‐derived β‐thiolactone (*N* ^Boc^‐PenTL) initiated by *N*‐Boc‐*L*‐cysteine methyl ester in *N*‐methyl‐2‐pyrrolidone (NMP) (Scheme 5).^[10]^ Both NMR spectroscopy and matrix‐assisted laser desorption ionization time‐of‐flight (MALDI‐ToF) mass spectrometry (MS) analyses of the resulting polymer corroborated the formation of *α‐*OMe,*ω*‐SH end‐capped PβTE. The molar mass of the PβTEs measured by SEC and ^1^H NMR analyses, yet relatively low (*M*_n_ <10,000 g.mol^−1^), was, to some extent, controlled by the monomer‐to‐initiator feed ratio (up to 100 : 1). However, the dispersity was relatively broad (*Đ*_M_=*ca*. 1.6–2.4), in part due to extensive transthioesterification reactions concomitant to the ROP. This easy thiol‐thioester exchange, often referred to as ‘dynamic nature of β‐thioesters’, also limits reaching high molar mass polymers. Due to the higher solubility of this PβTE in THF, the latter solvent was also examined as reaction medium. However, the ROP of *N* ^Boc^‐PenTL proved ineffective in THF, even under reflux, suggesting that NMP is essential for the polymerization to proceed. The authors proposed that the high polarity and low basicity of NMP increase the nucleophilicity of the thiol group of the initiator. Some mechanistic studies revealed that the propagating chain‐end is indeed the thiol group that attacks the carbonyl moiety to open the monomer ring by C(=O)−S bond scission. This is reminiscent of the anionic ROP of β‐lactones which can proceed via C(=O)−O bond ‘acyl’ cleavage leading to alkoxy propagating species and eventually an alcohol chain‐end (after final workup) (the other mode being C_β_−O(C(=O)) bond ‘alkyl’ cleavage, leading to carboxylate propagating species and eventually to a carboxylic chain‐end).^[11]^

**Scheme 6:**
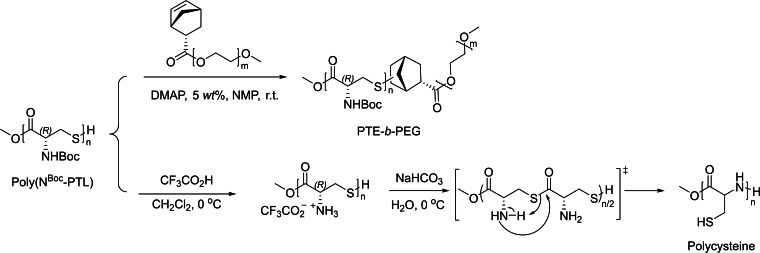
Post‐polymerization functionalization of a poly(β‐thioester) obtained from the ROP of a cysteine‐derived β‐thiolactone.^[10]^

Noteworthy, the thiol group exhibits a unique reactivity as compared to amino and hydroxyl groups. A famous example is the thiol‐ene click reaction which can be applied to modify thiol‐functionalized PTEs.^[12]^ Hence, reaction of the PβTE recovered from the ROP of the cysteine‐derived β‐thiolactone with a norbornene‐terminated poly(ethylene glycol) (PEG), using 4‐(*N*,*N*‐dimethylamino) pyridine (DMAP) as radical photoinitiator, quantitatively afforded the corresponding PTE‐*b*‐PEG block copolymer (Scheme 6, top). SEC monitoring showed the shift of the unimodal elution profiles to higher molar mass values throughout the reaction. Another valuable reaction is the intramolecular *S*‐to‐*N* acyl migration in the cysteine skeleton, triggered by the deprotection of the pendant *N*‐Boc groups, leading to the PβTE main chain transformation to polycysteine (Scheme 6, bottom). Surprisingly, the returned polycysteine displayed a higher molar mass than the starting PβTE, in spite of the abstraction of Boc groups that should ultimately decrease the value. The rationalization proposed relies on the higher rigidity of polycysteine – as the amide is more rigid than the thioester group due to the larger resonance between the carbonyl and the nitrogen‐, leading to a larger conformational shape of the macromolecules. Another suggestion, perhaps more likely, is the intermolecular bonding of polycysteines through the formation of S−S bonds.

**Scheme 7:**
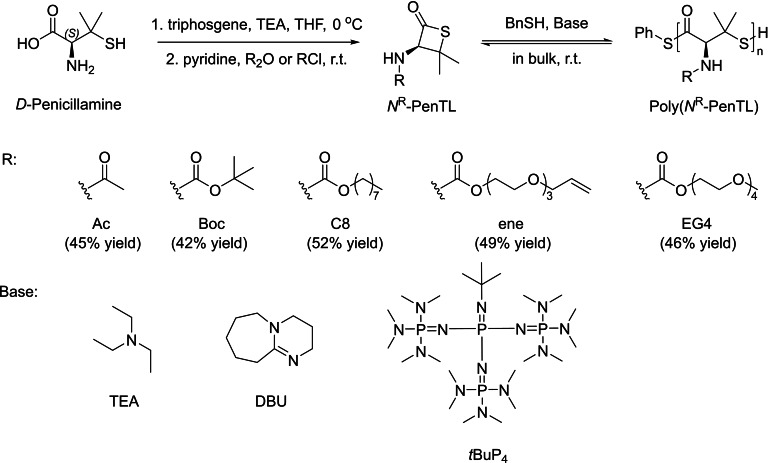
Synthesis and ROP of various N^R^‐PenTL type β‐thiolactones.^[13]^

To alleviate transthioesterification and tune more favorably the thermodynamics closer to equilibrium, the group of Lu recently modified the cysteine‐derived β‐thiolactone structure by introducing a geminal dimethyl group on the β position of the four‐membered ring (*N* ^R^‐PenTL). This monomer is easily synthesized from the naturally occurring amino acid *D*‐penicillamine in a one‐pot process and, notably, it can be tailored with different side‐chains.^[13]^ The bulk polymerization of *N* ^ene^‐PenTL promoted by a weakly basic organocatalyst such as triethylamine, was investigated in the presence of benzylthiol as co‐initiator at room temperature (Scheme 7). At a [*N* ^ene^‐PenTL]_0_/[NEt_3_]_0_/[BnSH]_0_ feed ratio of 100 : 1 : 1, 61% monomer conversion was achieved within 72 h, affording the corresponding PβTE with a considerably higher molar mass and narrower dispersity (*M*_n_=19,400 g.mol^−1^, *Ð*_M_=1.10) than the related PβTEs previously reported by the group of Matsuoka (same feed ratio; *vide supra*).^[10]^ Replacing triethylamine with the more basic 1,8‐diazabicyclo(5.4.0)undec‐7‐ene (DBU) accelerated the ROP with 59% monomer conversion being achieved within 6 h. Noteworthy, when the ROP of *N* ^ene^‐PenTL was quenched with iodoacetamide, MALDI‐ToF MS analysis revealed the formation of poly(*N* ^ene^‐PenTL) bearing PhCH_2_S− and −CH_2_CONH_2_ chain‐end groups, further confirming that thiolate is the propagating species. The molar mass of the poly(*N* ^ene^‐PenTL)s increased linearly from low (*M*_n_=6,400 g.mol^−1^, *Đ*_M_=1.11) to medium (*M*_n_=18,500 g.mol^−1^, *Đ*_M_=1.14) upon raising the feed ratio from 30 : 1 : 0.1 to 100 : 1 : 0.1. The *M*_n_ values also displayed a linear relationship with monomer conversion along with narrow dispersity, all supporting the controlled living character of the DBU‐catalyzed ROP of *N* ^ene^‐PenTL. The ROP of *N* ^C8^‐PenTL and *N* ^EG4^‐PenTL showed a similar control. The use of the more basic *t*BuP_4_ phosphazene superbase, allowed preparing higher molar mass poly(*N* ^C8^‐PenTL) (*M*_n_ up to 70,600 g.mol^−1^ using a feed ratio of 350 : 1 : 1), still maintaining a fairly narrow dispersity (*Đ*_M_=1.23). This provides a pathway to high molar mass PβTEs, which remains a bottleneck due to the dynamic nature of β‐thioesters.

**Scheme 8:**
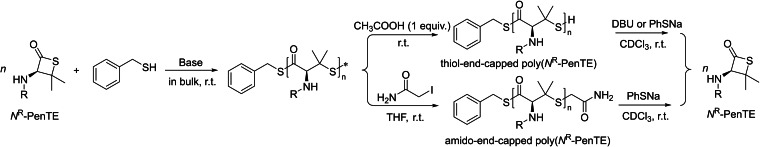
Facile chain‐end functionalization of various PTEs and subsequent depolymerization into *N* ^R^‐PenTE.^[13]^

The introduction of a geminal dimethyl group on the lactone ring, as a result of the Thorpe‐Ingold effect, considerably lowers the ring strain of the otherwise highly strained β‐thiolactone, and makes the recyclability of PβTEs based on such cysteine‐derived thiolactones possible.^[13]^ Hence, when the isolated *α*‐PhCH_2_S,*ω*‐SH‐poly(*N* ^R^‐PenTL) was treated with DBU at 65 °C, gradual depolymerization was confirmed by NMR and SEC analyses (Scheme 8). Yet, evaluation of the optical rotation of the recovered monomer revealed significant racemization upon depolymerization. More interestingly, at lower temperatures (such as room temperature), poly(*N* ^R^‐PenTL) could be completely depolymerized (>95% conversion) into the enantiopure monomers, revealing that a reduced temperature prevents racemization. On the other hand, *α*‐PhCH_2_S,*ω*‐CH_2_CONH_2_‐poly(*N* ^R^‐PenTL) obtained by reaction of the original polymer with iodoacetamide, remained unchanged upon treatment with DBU even after 12 h at room temperature. More interestingly, poly(*N* ^R^‐PenTL)s *ω*‐terminated with either −CH_2_CONH_2_ or −SH could be completely depolymerized into the *N* ^R^‐PenTL monomers when treated with PhSNa at ambient temperature. Hence, this class of monomers offers an appealing platform for environmentally‐friendly recyclable plastic materials within a circular economy.^[4]^

### 2.3 Ring‐opening polymerization of β‐thiobutyrolactone

**Scheme 9:**
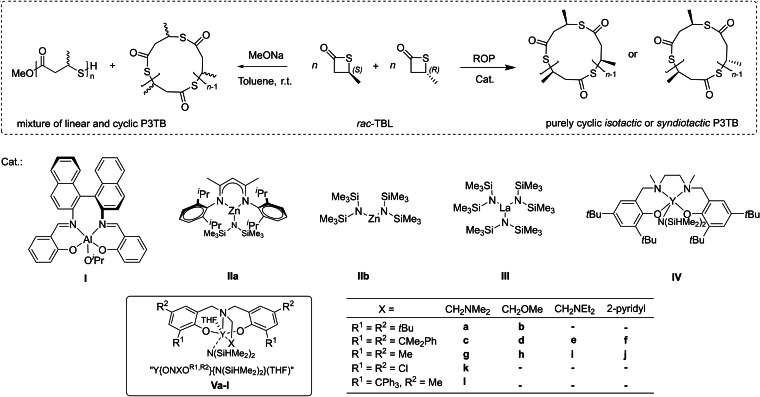
ROP of racemic β‐thiobutyrolactone (*rac*‐TBL) mediated by discrete yttrium catalysts towards stereoregular cyclic poly(3‐thiobutyrolactone) (P3TB).^[14]^

Very recently, our group uncovered the chemical synthesis of poly(3‐thiobutyrolactone) (P3TB) with controlled tacticity by the stereoselective ROP of *racemic* β‐thiobutyrolactone (*rac*‐TBL) (Scheme 9).^[14]^ P3TB, the thioester analogue of the ubiquitous poly(3‐hydroxybutyrate) (aka PHB), is a synthetic polythioester analogous to the isotactic one biosynthesized two decades ago from a recombinant strain of *E. Coli* cultured with thioalkanoic acids.^[7a]^ As exemplified above, the chemical synthesis of PβTEs by ROP of β‐thiolactones so far only involved organocatalysts and returned atactic PβTEs, unless starting from enantiomerically pure monomers (*vide supra*).

A variety of well‐established metal‐based catalysts/initiators was investigated into the ROP of *rac*‐TBL (Scheme 9). As anticipated, a simple highly nucleophilic initiator such as NaOMe smoothly enables the ROP of *rac*‐TBL at room temperature, yet, giving an ill‐defined mixture of cyclic and linear P3TB. Surprisingly, the zinc complexes (**II a** and **II b**), quite active with β‐lactones and *rac*‐LA,^[15]^ are inactive toward *rac*‐TBL. The chiral salen‐aluminium complex (**I**) and achiral salan‐yttrium complex (**IV**) lead to well‐defined cyclic P3TBs with significantly higher molar mass values than the theoretical ones, implying a poor initiating efficiency. The most promising results were obtained with yttrium complexes stabilized by tetradentate bis(phenolate) yttrium complexes “Y{ON(X)O^R1,R2^}” established by our group two decades ago.^[16]^ The ROP of *rac*‐TBL in toluene promoted by complex **V a**, a prototypical complex of this family flanked with simple *t*Bu substituents, proceeds very rapidly at ambient temperature, with turnover frequencies (TOF) that exceed 3,000 h^−1^; actually, the reaction proceeds even faster than the ROP of the parent oxo‐monomer, namely *rac*‐β‐butyrolactone (TOF=*ca*. 99 h^−1^),^[16e]^ under identical conditions. Microstructural studies by ^13^C NMR spectroscopy showed that all P3TBs formed feature syndio‐enriched microstructures (*P*_m_=0.31–0.37)^1^. The P3TBs also characterized by MALDI‐ToF MS analysis, revealed quite unexpectedly the formation of purely cyclic polymers. Regardless of the solvent (toluene or THF) and the nature of the co‐initiator (^*i*^PrOH, ^*i*^PrSH, or BnSH, 1 equiv. vs. Y), the experimental *m/z* values match the isotopic data calculated for [C_4_H_6_SO]_n_. Cyclic polymers are a special class of macromolecules; due to the constraints of the cyclic topology and the absence of chain‐ends, the properties of these macromolecules differ from their linear counterparts; for instance, they typically exhibit a higher density, higher glass transition temperature and melting point, lower intrinsic viscosity, increased crystallization rate, and higher refractive index.^[17]^ However, this has not yet been assessed with the P3TBs we recently prepared.

**Scheme 10:**
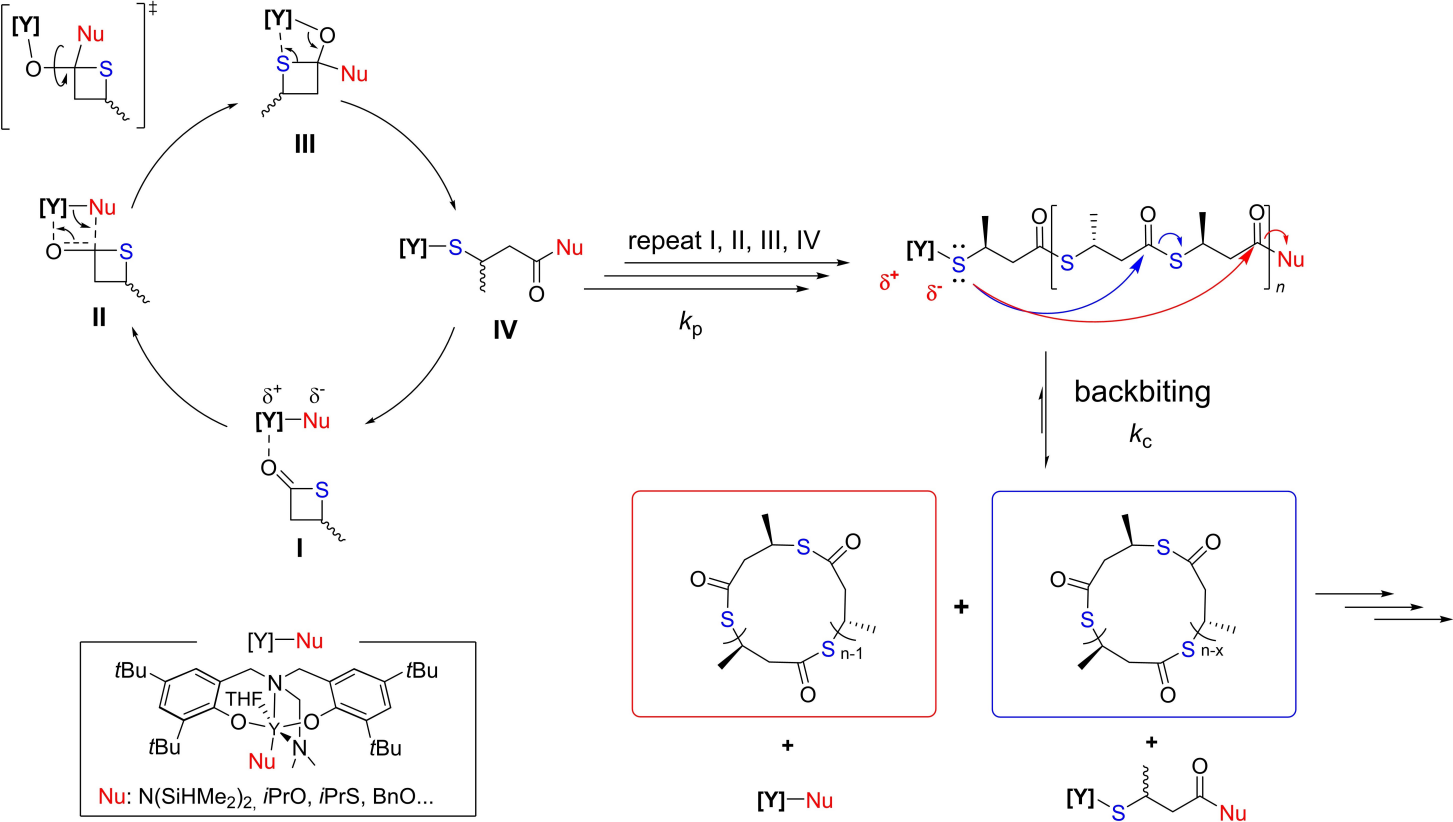
Proposed mechanism for the ROP of *rac*‐TBL promoted by complex **Va** with initiation, propagation, back‐biting, and ring‐expansion steps.^[14]^

Back‐biting occurs to a significant extent during the ROP of *rac*‐TBL promoted by these yttrium‐based catalysts. In fact, due to the intrinsically reactive dynamic thioester bonds within P3TB main chain and increased nucleophilicity of thiols *versus* alcohols, intramolecular transthioesterification occurs more readily than transesterification.^[18]^ As illustrated in Scheme 10, we assumed that thiolates attached to the yttrium metal center attack internal thioester groups at any site within the growing polymer chain, leading to the formation of cyclic P3TBs and ring‐expansion. In this model, high molar mass P3TBs are formed, implying a propagation rate significantly faster than the cyclization rate (*k*_p_≫*k*_c_, Scheme 10). On the other hand, common to chain‐growth polymerizations, chain transfer processes, such as back‐biting, can compete with chain propagation to enlarge the dispersity of the resulting polymer. In fact, the dispersity of P3TBs recovered from such yttrium‐promoted ROP reactions (*Đ*_M_=1.42–1.66) is definitively larger than those observed (*Đ*_M_=1.05–1.30) for poly(3‐hydroxybutyrate)s recovered from the ROP of *rac*‐β‐butyrolactone under similar conditions.[^16d^, ^16e^]

The most unique and valuable feature of “Y{ON(X)O^R1,R2^}” complexes is their ability to fine‐tune the stereocontrol of ROP. This can be done by simply changing the nature of the R^1^,R^2^ substituents on the phenolate moieties – most particularly of the R^1^ *ortho*‐substituents which are obviously the closest to the active center –, and by manipulating the side‐arm of the ligand (i. e. the pendant X donor group) which may affect the catalyst's electronics and/or sterics as well. Such tuning enabled accessing P3TBs with quite different levels of tacticity, with *P*_m_ values in the range 0.30–0.90. Yttrium complexes with moderately bulky *tert*‐butyl or cumyl‐substituted ligands give syndiotactic P3TB, which is line with previous works on the parent β‐butyrolactone and related derivatives.^[16]^ Unexpectedly, the catalyst system with the significantly larger trityl‐substituted ligand gave isotactic‐enriched P3TB (*P*_m_=0.58 at room temperature, *P*_m_=0.83 at −80 °C). Most unexpected too, the catalysts bearing the smaller substituents on the ligand also enabled to access isotactic P3TB. Hence, the simple “small/uncrowded” dimethyl or dichloro‐substituted catalysts offered highly isotactic P3TB when running the ROP in THF at low temperature (*P*_m_ up to 0.90). Mechanistically speaking, this behavior where chloro substituents apparently behave similarly to non‐bulky groups, differs from that of the same catalysts in the ROP of *rac*‐2‐alkoxymethylene‐β‐propiolactones where the electronic contribution of the Cl substituents is instrumental to establish H…Cl non‐covalent directing interactions that are essential to the observed isoselectivity.^[19]^

## 3 Ring‐opening polymerization of 5‐membered fused bicyclic γ‐thiolactones

**Scheme 11:**
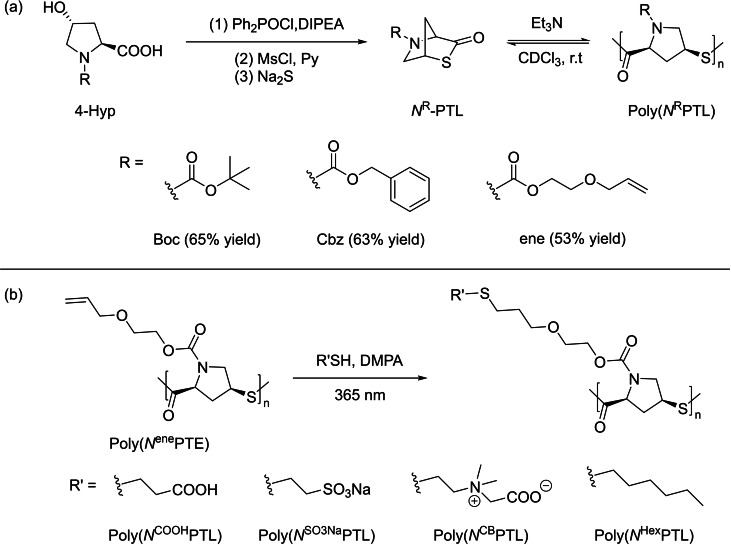
Synthesis and ROP of N^R^‐PTL and subsequent post‐polymerization functionalization of poly(N^R^PTL).^[20]^

In 2019, the group of Lu reported the controlled ROP of chiral *N*‐substituted *cis*‐4‐thia‐*L*‐proline thiolactone (*N* ^R^‐PTL, R=Boc, Cbz, or ene), initially synthesized from *trans*‐4‐hydroxy‐*L*‐proline (4‐Hyp), an abundant biosourced feedstock (Scheme 11).^[20]^ The authors rationalized the reactivity (TOF *ca*. 30 h^−1^ at room temperature) of this monomer by the high strain of the fused bicyclic thioester ring, as similarly observed for the bridged bicyclic γ‐butyrolactone.^[21]^ The ROP of *N* ^Boc^‐PTL performed by KO*t*Bu in highly polar solvents (*e. g*., acetonitrile, DMF or NMP) is uncontrolled and returned mixtures of both linear and small cyclic chains. Less polar solvents, such as chloroform and dichloromethane, suppress back‐biting reactions, achieving controlled ROP of *N* ^Boc^‐PTL at ambient temperature when using benzylthiol or triethylamine as the initiator and a catalyst, respectively. This afforded well‐defined poly(*N* ^Boc^‐PTL)s with narrow dispersity (typically *Đ*_M_=1.03–1.10) and predictable molar mass values (*M*_n_ up to 20,100 g.mol^−1^) dictated by the monomer‐to‐initiator feed ratio. These observations support a relatively fast initiation (as compared to propagation) along with limited undesirable side reactions (classically inter‐ and intra‐molecular transthioesterification reactions). Evidence of the terminal thiol group of the poly(*N* ^Boc^‐PTL)s was indirectly gained from its subsequent reaction with the iodoacetamide reagent, which returned *α*‐PhCH_2_S,*ω*‐CH_2_CONH_2_‐poly(*N* ^Boc^PTE) as revealed by MALDI‐ToF MS analysis. Switching the catalyst from triethylamine to the more basic DBU significantly accelerated the polymerization, thereby precluding kinetics monitoring. The ROP of *N* ^Cbz^‐PTL and *N* ^ene^‐PTL proceeded similarly to that of *N* ^Boc^‐PTL, highlighting the versatility of this polymerization.

The side‐chain allyl group of poly(*N* ^ene^PTL) can be easily modified upon reaction with various functional thiols under UV irradiation to give a set of chemically diverse PγTEs including carboxylic, sulfonate, zwitterionic, or alkyl groups. On the other hand, functionalization of poly(*N* ^Boc^PTL) with various thiols in the presence of triethylamine at room temperature did not proceed at the expense of smooth depolymerization back to the initial monomer (with quantitative regeneration of the monomer). This mild ROP/depolymerization chemistry may pave the way to sustainable recyclable materials. However, such PγTEs exhibit relatively low thermal stability and did not feature a melting temperature (*T*_m_) despite their chiral microstructure.

**Scheme 12:**
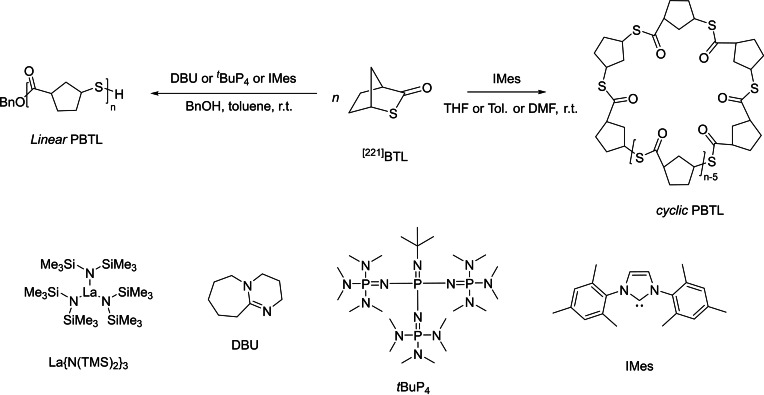
ROP of the bicyclic γ‐thiolactone ${}^{\left[221\right]}\mathrm{BTL}$ .^[22]^

To offer better promise to such PγTEs, *e. g*., to impart them with high crystallinity for targeted applications and also for better chemical recyclability, a bridged bicyclic thiolactone, namely 2‐thiabicyclo[2.2.1]heptan‐3‐one (${}^{\left[221\right]}\mathrm{BTL}$ ) was designed (Scheme 12). This latter monomer can be prepared in a racemic form from a bio‐based olefin carboxylic acid.^[22]^ The ROP of *rac*‐${}^{\left[221\right]}\mathrm{BTL}$ was first conducted effectively with four different catalyst systems: La{N(TMS)_2_}_3_, DBU and *t*BuP_4_ in the presence of benzyl alcohol (BnOH) as co‐initiator. Stereoregularity of these reactions yet significantly depended on the monomer concentration, reaction solvent polarity and monomer‐to‐initiator ratio. In toluene solution with [${}^{\left[221\right]}\mathrm{BTL}$ ]_0_ <1.6 g.mL^−1^ and [${}^{\left[221\right]}\mathrm{BTL}$ ]_0_/[BnOH]_0_ <300 : 1, at room temperature, all reactions gave stereo‐disordered PBTLs (*P*_r_ ∼0.21–0.46), regardless of the catalyst used. Unexpectedly, under these operating conditions, all these PBTLs exhibit a high crystallinity with *T*_m_ values typically in the range 166–176 °C. Increasing the monomer concentration ([${}^{\left[221\right]}\mathrm{BTL}$ ]_0_ >2.4 g mL^−1^) and decreasing the catalyst loading ([${}^{\left[221\right]}\mathrm{BTL}$ ]_0_/[*t*BuP_4_]_0_ >1000 : 1) returned essentially perfectly isotactic PBTL (*P*_r_=*ca*. 1), as further corroborated by a high *T*_m_ of 213 °C. Moving to *N*‐heterocyclic carbene (NHC), namely1,3‐bis(2,4,6‐trimethylphenyl)imidazol‐2‐ylidene (IMes), enabled to increase the feed ratio ([${}^{\left[221\right]}\mathrm{BTL}$ ]_0_/[IMes]_0_/[BnOH]_0_) up to 5000 : 1 : 1, and gave a highly stereoregular (*P*_r_=*ca*. 1), high molar mass (*M*_n,SEC_ = 48,300 g.mol^−1^), and crystalline PBTL with *T*_m_=213 °C. In line with previous observations by Waymouth^[23]^ which showed that, with the help of an alcohol, NHCs are able to promote ROP of cyclic esters to produce linear polyesters, the ROP of ${}^{\left[221\right]}\mathrm{BTL}$ mediated by IMes in the presence of alcohol initiators generated well‐defined linear PTEs. Remarkably, the ROP of ${}^{\left[221\right]}\mathrm{BTL}$ by NHC without alcohol gives cyclic PBTLs, as evidenced by MALDI‐ToF MS analysis, and further demonstrated by its lower intrinsic viscosity compared with its linear counterpart (typically [η]_cyclic_/[η]_linear_=*ca*. 0.7).[^17a^, ^17b^] The melting temperature of PBTLs increases monotonously and linearly with the tacticity (*T*_m_=54.8×*P*_r_+157.4 °C with *P*_r_ ranging from 0.47 to 1). These results show that these PTEs exhibit a singular ability to crystallize, even with a high degree of stereochemical disorder. In addition, the thermal stability of cyclic PBTL is about 7 °C higher than that its linear counterpart (*T*_d_=321 °C), which is consistent with the fact that the thermal stability of cyclic polymers is generally higher than that of the linear analogues.[^17a^, ^17b^] This provides a valuable thermal processing window of more than 100 °C for PBTL (above the *T*_m_ and below the *T*_d_).

Another valuable property of these PBTLs is their intrinsic chemical recyclability for a closed‐loop life cycle. When cyclic PBTL was treated with either La{N(TMS)_2_}_3_ at 100 °C in bulk, or with IMes (2 M toluene solution) at room temperature, depolymerization occurred quantitatively giving back the monomer without isomerization. The recycled ${}^{\left[221\right]}\mathrm{BTL}$ can then be directly repolymerized into PBTL; this was assessed on one cycle but can, in principle, be extended to multiple recycling. No information was provided on the possibility to depolymerize/recycle the linear polymer. Overall, these PBTL polythioesters possess a unique set of desired properties, such as tunable tacticity from atactic to perfect stereoregularity, intrinsic tacticity‐independent crystallinity, high thermal stability and chemical recyclability.

## 4 Ring‐opening copolymerization of cyclic thioanhydrides or thiobutyrolactone with episulfides and epoxides

Aliphatic polythioesters are commonly prepared by ROP of the corresponding cyclic thiolactones. Nevertheless, particularly at high monomer conversion, the ROP of thiolactones can be undermined by detrimental side reactions such as transthioesterification. Additionally, the resulting PTEs exhibit a limited range of properties due to restricted functional diversity of thiolactones and the lack of post‐polymerization functionalization. An alternative synthetic pathway is the alternating copolymerization of cyclic thioanhydrides with epoxides and/or episulfides.

### 4.1 Ring‐opening copolymerization of cyclic thioanhydrides and episulfides

**Scheme 13:**
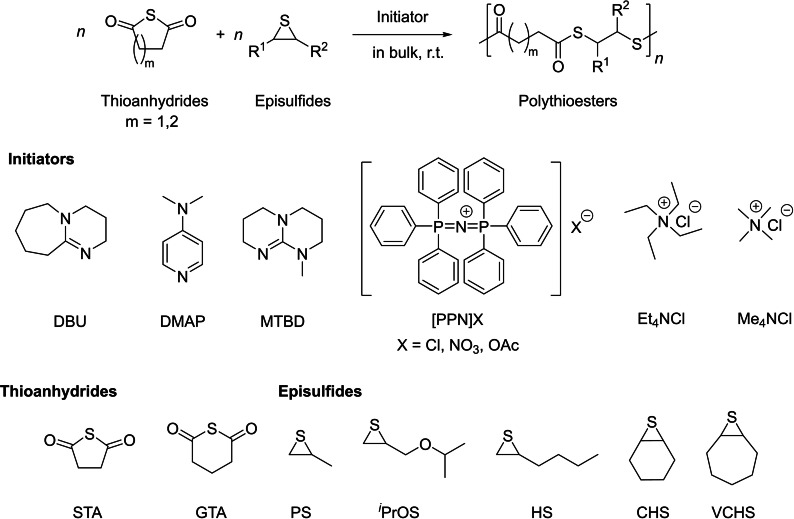
ROCOP of cyclic thioanhydrides with episulfides towards diverse aliphatic polythioesters.^[24]^

In this regard, the group of Lu reported the synthesis of structurally diverse PTEs by ring‐opening copolymerization (ROCOP) of cyclic thioanhydrides and episulfides (Scheme 13).^[24]^ The ROCOP of succinic thioanhydride (STA) and propylene sulfide (PS) mediated by organobases, such as DBU, DMAP, and 7‐methyl‐1,5,7‐triazabicyclo(4.4.0)dec‐5‐ene (MTBD), gives rise to the corresponding PTEs; yet, the polymers featured significantly lower molar mass values than the theoretical ones, even at high STA conversion (*ca*. 85%). Switching the organobase by the organic ammonium salt [PPN]Cl ([PPN]=bis(triphenylphosphine)iminium), the ROCOP of STA and PS at a [PS]_0_/[STA]_0_/[PPN]_0_ feed ratio of 1000 : 250 : 1, led to the corresponding PTE with a high molar mass (*M*_n,SEC_=35,200 g.mol^−1^), in good agreement with the theoretical value (*M*_n,theo_=37,100 g.mol^−1^) along with narrow dispersity (*Đ*_M_=1.24). However, side reactions, such as transthioesterification, occurred at high monomer conversion (*ca*. 99%), leading to PTEs with broader dispersity (*Đ*_M_=1.72). Using an initiator with a nucleophilic anion, such as in [PPN]OAc, resulted in improved performance in terms of polymerization rate and controlled character (*Đ*_M_ <1.30). The molar mass of the resulting PTEs could also be tuned by the addition of a chain‐transfer agent, such as benzyl mercaptan (BnSH). Another notable feature of this methodology is its versatility towards different thioanhydrides and episulfides, giving the corresponding alternating PTEs with various functionalities (Scheme 13). Poly(STA‐*alt*‐PS) thus prepared is a crystalline material (*T*_m_=80 °C) with a refractive index (*n*_D_) of 1.78 which is the highest value among the reported sulfur‐containing polymers.^[24]^

### 4.2 Ring‐opening copolymerization of cyclic thioanhydrides and epoxides

**Scheme 14:**
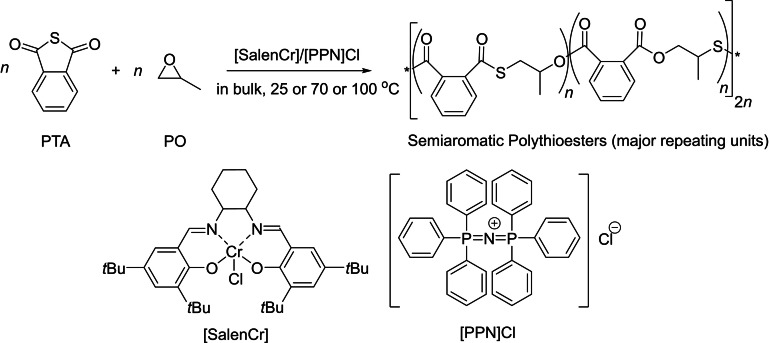
ROCOP of phthalic thioanhydride (PTA) with propylene oxide (PO) towards semi‐aromatic poly(thioester‐*alt*‐ester)s.^[25a]^

The ring‐opening copolymerization of cyclic thioanhydrides and epoxides is another attractive approach to access PTEs. The group of Lu reported the ROCOP of phthalic thioanhydride (PTA) and propylene oxide (PO) mediated by a salen‐Cr complex in conjunction with [PPN]Cl (Scheme 14).^[25a]^ The reactions proceeded with high activity (TOF up to 1,420 mol(monomer).mol(Cr)^−1^.h^−1^ at 100 °C in bulk). However, the copolymers produced at high PTA conversions under these conditions contained not only the desired polythioester and polyester segments, but also polythioether and polyether segments, as characterized by NMR spectroscopy. At 25 °C, the resulting polymer contained up to 98% thioester‐ester linkages with a few thioether‐ester linkages; but at 100 °C, thioester‐ester linkages dropped to ca. 82%, with up to 5% ester‐ester and 13% thioether‐ester linkages, due to significant intra‐ and intermolecular transesterifications. In this regard, developing an efficient approach to suppress these side reactions is highly desirable.

**Scheme 15:**
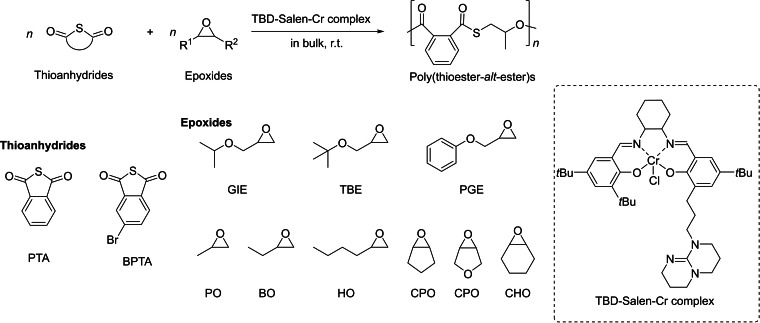
ROCOP of cyclic thioanhydrides with epoxides towards poly(thioester‐*alt*‐ester)s mediated by an acido‐basic cooperative catalyst.^[25b]^

The authors suggested that the relatively lower reactivity of PTA as compared to cyclic thioesters leads to transesterification reactions and thereby to dispersed PTEs with variable chemical sequences. Subsequently, a bifunctional Cr‐based complex tethered with the organobase 1,5,7‐triazabicyclo[4.4.0]dec‐5‐ene (TBD) was used as the catalyst to induce the ROCOP; the reactions conducted in bulk thus gave well‐defined poly(thioester‐*alt*‐ester)s with completely alternating structures, controlled molar mass, and low dispersities (*Đ*_M_=1.18–1.25) (Scheme 15).^[25b]^ Electrospray ionization mass spectrometric analysis (ESI MS) in combination with NMR and DFT studies suggested that the Cr centre activates the epoxide and that TBD activates PTA, then facilitating the alternating insertions. The reaction was successfully extended to a variety of epoxides.

### 4.3 Ring‐opening copolymerization of cyclic thioanhydrides, anhydrides and epoxides

**Scheme 16:**
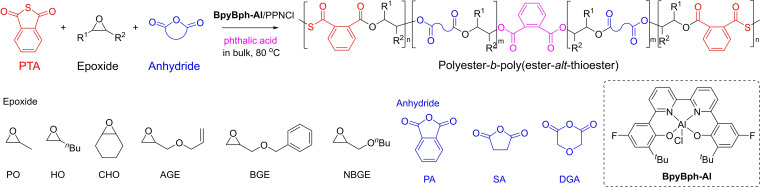
One‐step ROCOP of cyclic thioanhydride, epoxide and cyclic anhydride mixtures towards sequence‐controlled polyester‐*b*‐poly(ester‐*alt*‐thioester).^[26]^

In 2022, the group of Li subsequently established an effective pathway to achieve the ROCOP of cyclic anhydrides, thioanhydrides and epoxides mixtures, giving sequence‐controlled polyester‐*b*‐poly(ester‐*alt*‐thioester)s (Scheme 16).^[26]^ The ROCOP of PTA and propylene oxide (PO) was used as a model reaction to screen the catalysts; the binary system made of the salen‐like complex BpyBph‐Al and [PPN]Cl stands out in terms of activity and limited transthioesterification side reactions at high temperature (such as 80 °C) in bulk, although the molar mass values of the resulting copolymers were significantly lower than the theoretical ones. The ROCOP of PTA, 1‐hexene oxide (HO) and phtalic anhydride (PA) was performed with this binary catalyst system in the presence of a chain transfer agent, such as phthalic acid, at 80 °C in bulk. Due to the significantly different reactivity of anhydride and thioanhydride towards alkoxide chain‐end, this led to the formation of polyester‐*b*‐poly(ester‐*alt*‐thioester)s, as thoroughly characterized by NMR spectroscopy. This one‐step, three‐component copolymerization approach was applied to a variety of epoxides and anhydrides with PTA, giving well‐defined sequence‐controlled block copolymers without transesterification.^[26]^ This chemoselective copolymerization provides a straightforward and powerful approach to PTEs with structural diversity for different applications.

**Scheme 17:**
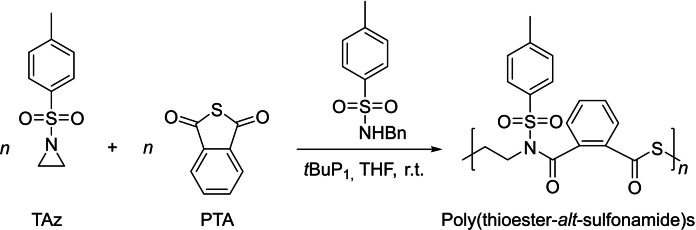
ROCOP of *N*‐tosylaziridine (TAz) with phthalic thioanhydride (PTA) towards poly(thioester‐*alt*‐sulfonamide)s.^[27]^

Poly(ester amide)s with both esters and amides in the polymer backbone have attracted increasing attention, due to the combination of biodegradability/biocompatibility of polyesters with excellent thermal and mechanical performances of polyamides. The substitution of ester groups of poly(ester amide)s with thioester groups and of the amide by sulfonamide groups, respectively, leading to the formation of poly(thioester sulfonamide), may expand the scope of the applications of poly(ester amide)s. With these considerations in mind, the group of You described the preparation of poly(thioester sulfonamide) by ROCOP of *N*‐tosylaziridine (TAz) with PTA promoted by the phosphazene *t*BuP_1_ in the presence of *N*‐benzyl‐4‐methylbenzenesulfonamide (BnN(H)Ts) (Scheme 17).^[27]^ Analysis by NMR and MALDI‐ToF MS revealed the formation of mixtures of the expected poly(thioester‐*alt*‐sulfonamide) copolymers and of the homopolymer of TAz. By increasing the [PTA]_0_/[TAz]_0_ ratio to 2.5 : 1, well‐defined alternating copolymers were selectively generated with controlled molar mass (*M*_n,SEC_ up to 7,300 g.mol^−1^) and low dispersity (*Đ*_M_ <1.13). Taking advantage of the mild catalytic process and living character of the polymerization, novel triblock copolymers, such as poly(TAz‐*alt*‐PA)_20_‐*b*‐PTAz_16_‐*b*‐poly(TAz‐*alt*‐PTA)_22_ with a *T*_g_ of 104 °C were synthesized.

### 4.4 Ring‐opening copolymerization of thiobutyrolactone with epoxides

**Scheme 18:**
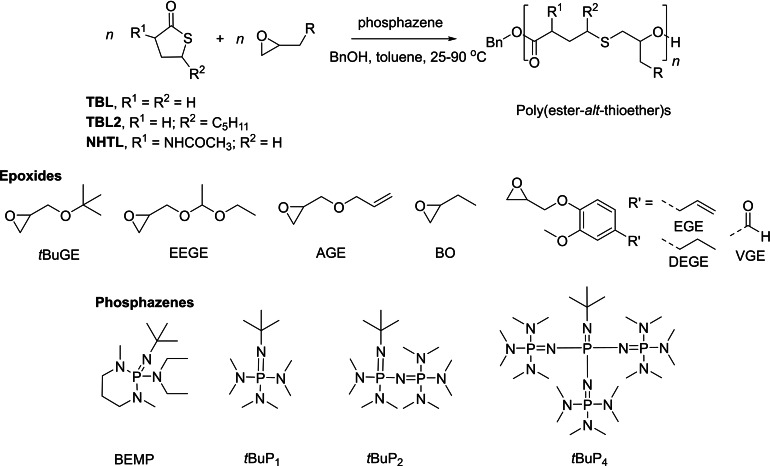
Ring‐opening copolymerization of γ‐thiobutyrolactones with functional epoxides towards poly(ester‐*alt*‐thioether)s.^[28]^

γ‐Thiobutyrolactone, the sulfur analog of γ‐butyrolactone, due to its low strain energy, is commonly referred to as ‘non‐polymerizable’. Yet, introduction of a high‐strain comonomer into the ‘non‐polymerizable’ reaction system can change the free enthalpy of the system, perturb the propagation/depropagation equilibrium continuously and shift it towards propagation. Hence, the group of Illy investigated the ROCOP of γ‐thiobutyrolactone and functional epoxides such as *tert*‐butyl glycidyl ether (*t*BuGE) by the combination of a phosphazene such as *t*BuP_4_ and BnOH; however, unexpectedly, they recovered poly(ester‐*alt*‐thioether)s instead of the anticipated poly(ester‐*alt*‐thioester)s (Scheme 18).^[28a]^ Upon running the polymerization at rather low temperatures (50 °C or even better at 35 °C), the ROCOP featured controlled characteristics: the *M*_n_ values increased linearly with the monomer conversion, with narrow dispersity (*Đ*_M_ <1.22). The methodology was successfully extended to the preparation of higher, renewable linear poly(ester‐*alt*‐thioether)s made with epoxides bearing different side chains, and bio‐based *N*‐acetylhomocysteine thiolactone (NHTL), by using 2‐*tert*‐butylimino‐2‐diethylamino‐1,3‐dimethylperhydro‐1,3,2‐diazaphosphorine (BEMP) ‐BnOH as initiating system in THF (Scheme 18).[^28b^, ^28c^]

**Scheme 19:**
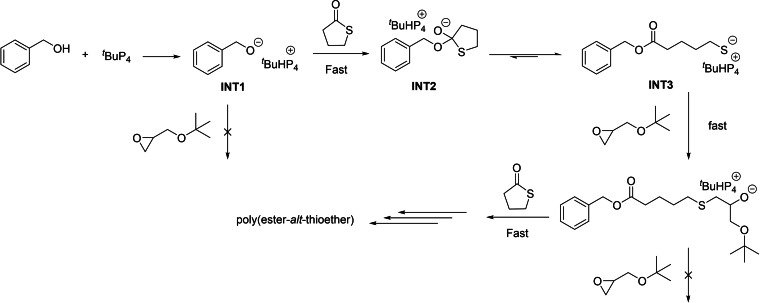
A plausible alternative mechanism for ROCOP of γ‐thiobutyrolactone with an epoxide.^[28a]^

The authors proposed a mechanism accounting for the formation of poly(ester‐*alt*‐thioether)s (Scheme 19). It involves a fast complexation between the generated phosphazenium alkoxides (**INT1**) and thiolactone, preventing the homopolymerization of epoxide. The generated intermediate (**INT2**) is in equilibrium with the thiolate‐terminated active chain (**INT3**), which immediately reacts with epoxide, leading to the alternated polymer. The bulky *t*BuP_4_ associated with the terminated chain end presumably create congested circumstances at the terminal group which prevent back‐biting.^[28a]^

### 4.5 Ring‐opening copolymerization of selenobutyrolactone with episulfides

**Scheme 20:**
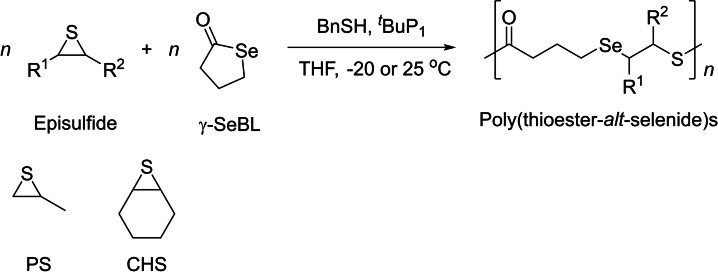
ROCOP of episulfides with γ‐selenobutyrolactone (γ‐SeBL) towards alternating poly(thioester‐*alt*‐selenide)s.^[29]^

Selenium is an element with a relatively large atomic radius and a lower electronegativity than sulfur and oxygen. Se‐containing polymers have found a variety of applications in biomedical and adaptive materials. However, methodologies for the incorporation of Se atoms into a polymer backbone are still rare. In this context, the group of Zhang reported the ROCOP of episulfides and γ‐selenobutyrolactone (γ‐SeBL), yielding the corresponding poly(thioester‐*alt*‐selenide)s (Scheme 20).^[29]^ The ROCOP of propylene sulfide (PS) and γ‐SeBL was investigated by the combination of the *t*BuP_1_ phosphazene and BnSH at −20 °C in THF; it efficiently gives the corresponding copolymers (TOF up to 1,200 h^−1^), with relatively narrow dispersity (*Đ*_M_=1.2‐1.4) and molar mass (*M*_n_) up to 12,800 g.mol^−1^. The recovered copolymers were primarily the expected alternating poly(thioester‐*alt*‐selenide)s, yet along with several other populations resulting from transthioesterification and formation of S−S and S−Se bonds *via* oxidation upon air‐exposure. These poly(thioester‐*alt*‐selenide)s exhibit a remarkably high refractive index up to 1.74, of interest for potential applications in optical materials. The ROCOP of γ‐SeBL and cyclohexene sulfide (CHS) at room temperature leads to the corresponding rigorously alternating PTE.

## 5 Ring‐opening polymerization of 6‐membered δ‐thiolactones and derivatives

### 5.1 Ring‐opening polymerization of δ‐thiovalerolactone

**Scheme 21:**
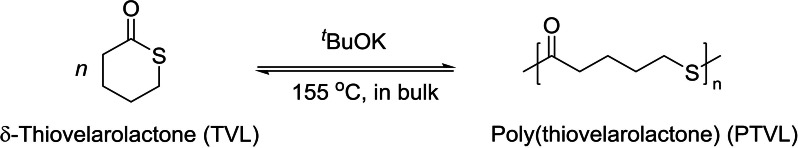
Early attempts at ROP of δ‐thiovalerolactone.^[30]^

Early attempts on ROP of the 6‐membered δ‐thiovalerolactone (TVL) date back to 1968 (Scheme 21);^[30]^ yet, the monomer conversion was extremely low (*ca*. 21%) and molar mass and dispersity data of the resulting PTVL remained limited, at that time. The authors hypothesized that there is a monomer/polymer equilibrium (that is quite usual in the field of heterocyclics ROP),[^11b^, ^31^] leading to a limited molar mass.

### 5.2 Ring‐opening (co)polymerization of thiolactide

**Scheme 22:**
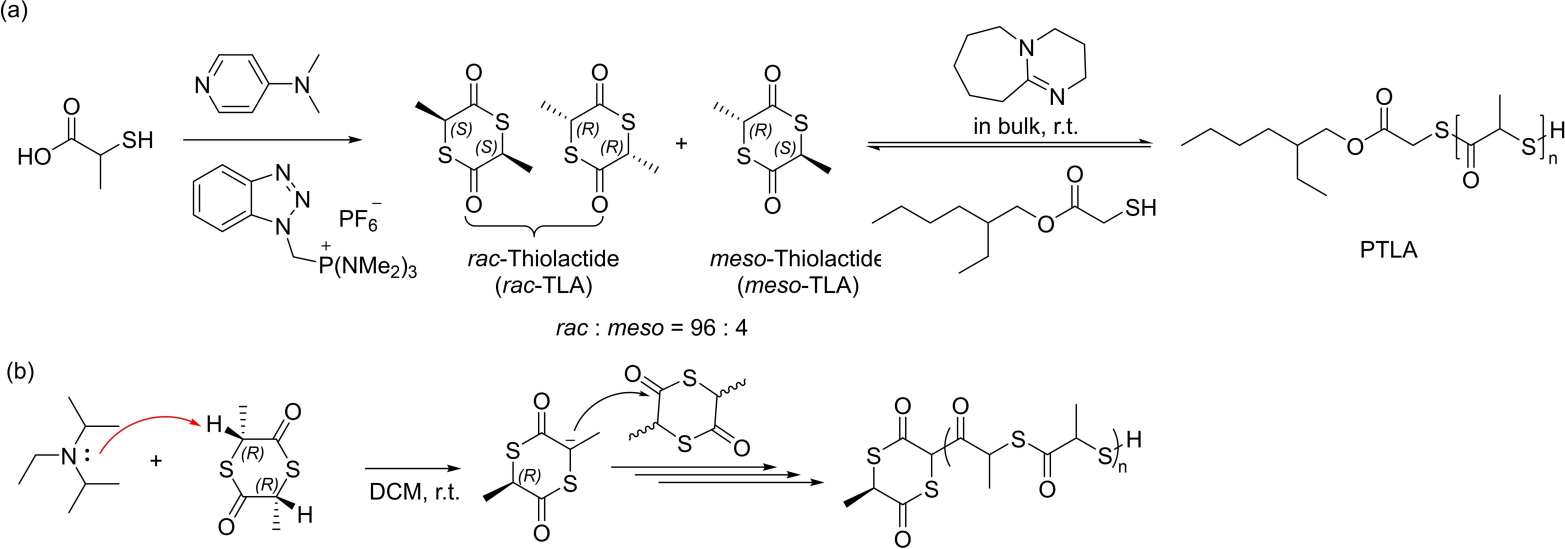
(a) The synthesis of *rac*‐thiolactide and its ROP by a basic organocatalyst; (b) ROP of *rac*‐TLA initiated by a non‐nucleophilic amine.^[32]^

The group of Matsuoka reported the formation of polythiolactide (PTLA), an attractive analogue of the ubiquitous polylactide (PLA) – an extensively studied renewable and biodegradable polymer (Scheme 22, top).^[32]^ *Racemic* thiolactide (*rac*‐TLA) had been synthesized analogously to lactide prior to this work, that is by condensation of the material obtained from thermolysis of PTLA, while cyclization reaction from α‐mercapto acid was implemented in this contribution similar to that of Tao's findings (*vide infra*). The isolated TLA consists of the *racemic* and *meso* forms at a ratio of 98 : 2–95 : 5. DFT calculations combined to the condensation of cyclic (*R*)‐thiolactic acid suggested that the *racemic* form is much more stable than the *meso* isomer, as also demonstrated by the group of Tao (*vide infra*).^[33]^ The ROP of *rac*‐TLA examined with a thiol/DBU combination at room temperature showed first that the conversion of the monomer remained limited to 25%, even in concentrated solutions, or by extension of the reaction time or by increasing the reaction temperature. The monomer conversion reached about 50% when performing the ROP in bulk, but still did not increase upon prolonging the reaction time. Also, decreasing the amount of the initiator increased the molar mass of the PTLA at constant monomer conversion. These findings suggest that the ROP of TLA reaches an equilibrium between monomer and polymer, which was further confirmed by three different post‐polymerization treatments: i) additional feeding of *rac*‐TLA resulted in the same ceiling conversion along with an increase of the molar mass of the resulting PTLA, ii) heating to 60 °C led to a decrease of the monomer conversion, and iii) addition of CH_2_Cl_2_ gave rise to partial depolymerization to TLA. Surprisingly, sterically hindered bases, such as diisopropylethylamine or 1,2,2,6,6‐pentamethylpiperidine, were found to promote ROP without addition of an initiator, while a weaker amine, such as pyridine, was unable to do so. The authors presumed that these non‐nucleophilic amines enable deprotonation at the α‐position of the thioester carbonyl group thus initiating the polymerization (Scheme 22, bottom); this further suggests that thioester has a more acidic carbonyl α‐proton than its oxoester analogue. PTLA was always isolated as a mixture of linear and cyclic polymers, as verified by MALDI‐ToF MS analysis. In fact, intra‐ and intermolecular thiol‐thioester exchange reactions occur during the ROP.

**Scheme 23:**
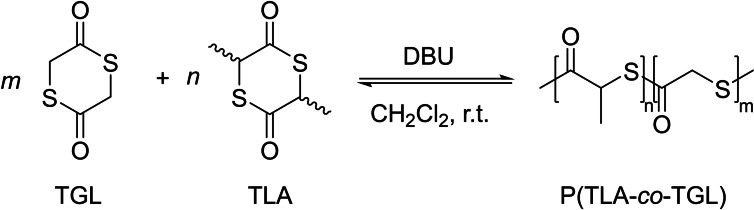
Ring‐opening copolymerization of thioglycolide and thiolactide.^[32]^

The copolymerization of *rac*‐thiolactide (TLA) with thioglycolide (TGL) was also briefly studied (Scheme 23).^[32]^ Prior investigation of the homopolymerization of TGL using a combination of thiol and DBU also reached an equilibrium as observed in the ROP of *rac*‐TLA. The ring‐opening copolymerization of TGL with *rac*‐TLA proceeds with higher conversions of both monomers as compared to those reached in the corresponding homopolymerizations; no detail on reactivity ratios, microstructure and properties of these copolymers were however reported.

### 5.3 Ring‐opening polymerization of substituted 6‐membered thiolactones

**Scheme 24:**
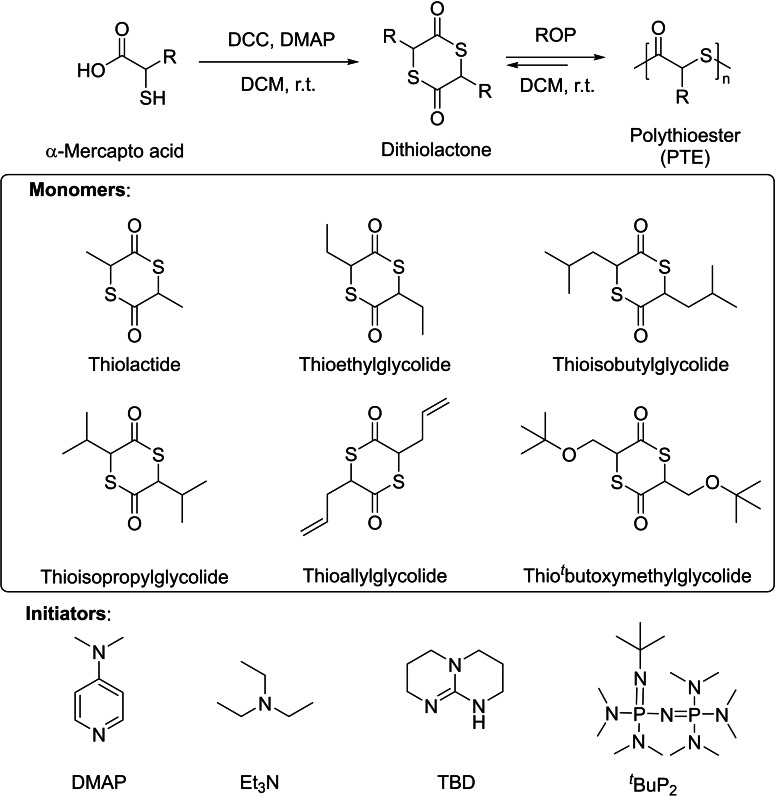
Synthetic route and ROP of dithiolactones by basic organocatalysts.^[33]^

In 2021, the group of Tao revisited the chemistry of PTLA, also expanding the monomer scope to different substituted 6‐membered thiolactones (Scheme 24).^[33]^ The latter monomers bearing different side chains were efficiently synthesized by cyclization reaction from α‐mercapto acid in good yields. Yet, the isomeric (*rac*/*meso*) ratio of the monomers was not documented. Investigation of the thermodynamics of the ROP of *rac*‐TLA indicated that the ceiling temperature is −67 °C, and 197 °C for an initial monomer concentration of 1.0 and 5.0 M, respectively; this suggests that ROP of *rac*‐TLA should reach complete conversion rather easily when conducted at high concentration. In fact, the ROP of *rac*‐TLA promoted by a basic organocatalyst such as DMAP, in the presence of benzyl thiol as co‐initiator, proceeds rapidly in a controlled fashion at 25 °C in dichloromethane at high monomer concentration ([TLA]_0_=5.9 M), achieving 58% conversion in 10 min. The ROP reaction under these conditions is rather well‐controlled, as confirmed by the linear growth of the experimental molar masses (*M*_n,SEC_) as a function of monomer conversion, coupled with rather narrow dispersities (*Đ*_M_ <1.40). The presence of a terminal thiol moiety was demonstrated by the end‐capping with iodoacetamide, which afforded a PTLA bearing PhCH_2_S− and −CH_2_CONH_2_ chain‐end groups. However, inevitable transthioesterification side reactions occurred during polymerization, as demonstrated by MALDI‐ToF MS analysis (observation of half TLA repeating units). Of note, the ROP of *rac*‐TLA using DMAP or benzyl thiol alone, under comparable conditions, was ineffective. Collectively, all these results suggest an activation mechanism where DMAP activates the benzyl thiol initiator or thiol chain‐end. Switching the DMAP catalyst to triethylamine or stronger bases, namely 1,5,7‐triazabicyclo[4.4.0]dec‐5‐ene (TBD, p*K*_a_^CH3CN^=26.0) or a phosphazene such as *t*BuP_2_ (p*K*_a_^CH3CN^=33.5), improved the polymerization rate but in an uncontrolled manner (*Đ*_M_ >1.50); this corroborates that extensive transthioesterification competes with chain propagation with such strongly basic organocatalysts.

As aforementioned, a remarkable feature of the obtained PTLA is its chemical recyclability due to its low ceiling temperature. The group of Tao further evidenced quantitative recovery of the TLA monomer in high selectivity within a few minutes at room temperature via ring‐closing depolymerization, with or without solvent.^[33]^ It returned a mixture of *rac*/*meso*‐TLA in a *ca*. 98 : 2 ratio, in line with works and DFT calculations from the group of Matsuoka,^[32]^ irrespective of the severe extent of transthioesterification reactions; pure *rac*‐TLA could be further separated by recrystallization in 95% isolated yield. Collectively, owing to the large energy difference between the *rac* and *meso* isomers of TLA, coupled with the increased acidity of the α‐carbonyl H (as compared to the parent lactide), depolymerization of PTLA is accompanied by the simultaneous quantitative *meso*‐to‐*rac*‐TLA conversion, thereby ensuring high depolymerization selectivity.

Another notable contribution from the group of Tao is to provide opportunities to implement ROP of related 6‐membered dithiolactones bearing different side chains; this enabled tailoring of physical, mechanical and biological properties of the resulting PTE materials.^[33]^ The ROP of these functional dithiolactones was successfully achieved under the reaction conditions established for TLA, and proceeded in a controlled manner. All the resulting PTLAs could be depolymerized into the corresponding monomers. The thermal properties of these polythioesters spanned a broad glass transition and degradation temperature range (*T*_g_=−19.2 to +1.9 °C and *T*_d_^5%^=182–254 °C; *T*_d_^5%^ defined as the temperature for 5% weight loss, respectively); unexpectedly, the atactic poly(thioisopropylglycolide) is semicrystalline with a *T*_m_ of 115.6 °C. Interestingly, a random copolythioester made from *rac*‐TLA and *rac*‐thioethylgylcolide was found to depolymerize easily as well, yet providing, as expected, a mixture of symmetrical and asymmetrical dithiolactone monomers. Hence, overall, the DMAP‐catalyzed ROP of dithiolactones paves a new way towards functional, recyclable PTEs from readily available feedstock, foreseeing the development of new polymer materials.

**Scheme 25:**
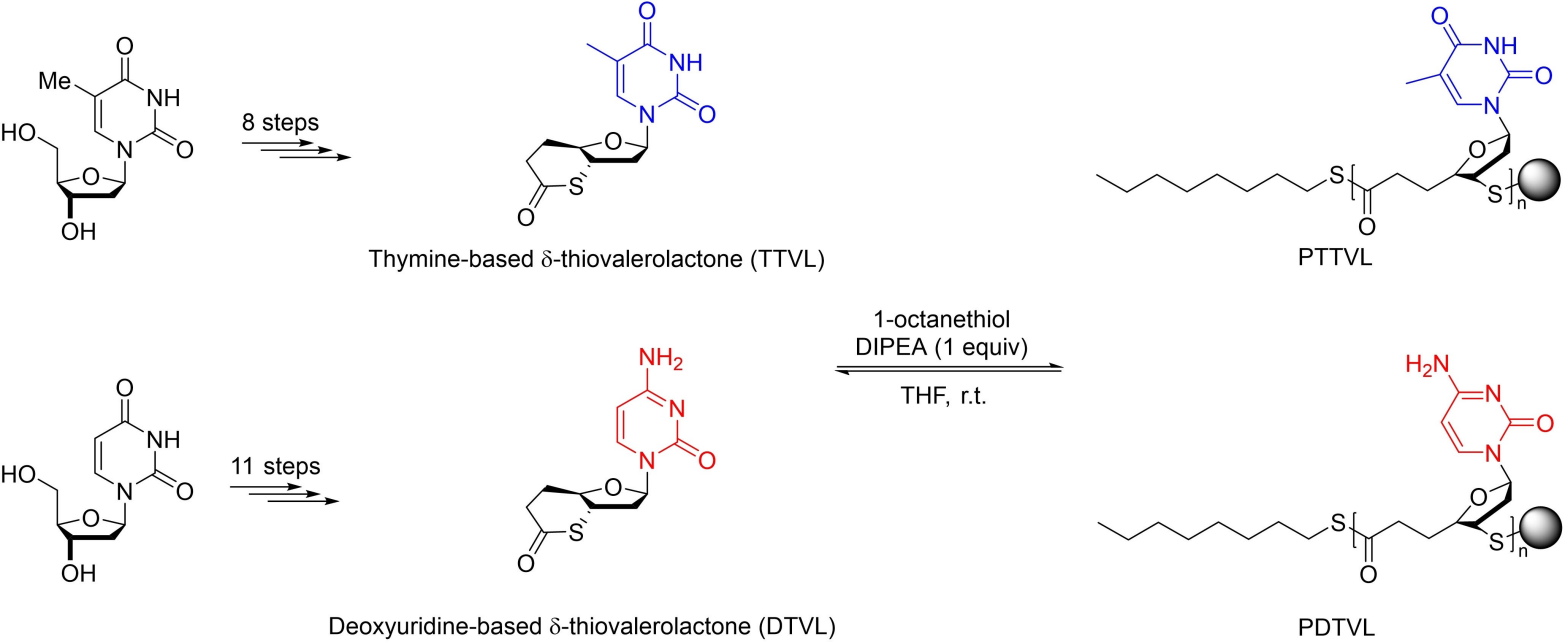
ROP of nucleobase‐functionalized δ‐thiolactones.^[34]^

In 2018, the group of Bowman reported the ROP of 6‐membered δ‐thiovalerolactones functionalized with natural nucleosides into the corresponding functional poly(δ‐thiovalerolactone)s (Scheme 25).^[34]^ Due to the structural and stereochemical similarity to DNA of this latter linked‐DNA thioester, this non‐natural polymer is anticipated to tightly bind to its native complement (i. e., native DNA); it thus constitutes a first step towards a platform that is expected to culminate in the synthesis of sequence‐controlled polymers via dynamic‐template‐directed synthesis. The targeted δ‐thiolactones, that is thymine‐based thiovalerolactone (TTVL) and deoxyuridine‐based thiovalerolactone (DTVL), were synthesized in multiple steps, and subsequently subjected to ROP mediated by basic organocatalysts. TTVL, used as a model monomer, undergoes ROP in the presence of *N*,*N*‐diisopropylethylamine (DIPEA) as catalyst and 1‐octanethiol as initiator, in THF at room temperature. The highest molar mass (up to 5,700 g.mol^−1^) of PTTVL was achieved at a monomer‐to‐initiator ratio of 100 : 1 : 1 over prolonged reaction time (6 h); yet, the monomer conversion was relatively low (*ca*. 27%). *In situ* end‐capping of these polymers following the ROP reaction was found to be critical, likely due to their low ceiling temperature; otherwise, significant degradation or inconsistent results were noted, indicating that such a system exhibits an intrinsic reversible monomer‐polymer equilibrium. The optimized ROP conditions were extended to DTVL, giving a PDTVL oligomer (*M*_n,SEC_ up to 2,700 g.mol^−1^). These general conditions also provided low molar mass copolymers of TTVL and DTVL (*M*_n_=3,700 g.mol^−1^, *Đ*=1.45). Interestingly, the thiol‐thioester exchange reaction was demonstrated by (i) the reaction of 1‐octanethiol with the isolated PδTE, and by (ii) the coalescing of two uncapped homopolymers of different chemical composition into a single copolymer; these observations strongly support the dynamic and responsive behavior of PTEs.

Overall, although the scope of 6‐membered thioesters has not been extensively studied, these few examples demonstrate its large potential as recyclable materials due to the low ceiling temperature.

## 6 Ring‐opening polymerization of 7‐membered ϵ‐thiocaprolactone

**Scheme 26:**
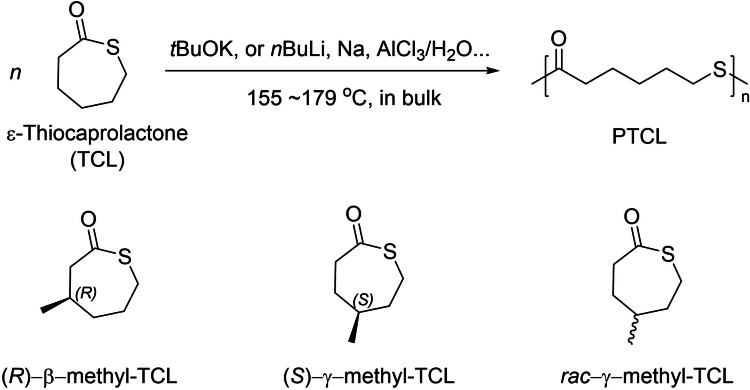
ROP of ϵ‐thiocaprolactone (TCL) initiated by inorganic bases.[^30^, ^35^]

The initial report of the ROP of ϵ‐thiocaprolactone (TCL) and β/γ‐methyl‐substituted ϵ‐thiocaprolactone dates back to 1964 (Scheme 26).[^30^, ^35^] These early polymerizations were promoted by inorganic bases such as *t*BuOK, *n*BuLi, Na dispersion, and they were most likely uncontrolled with limited information on molar mass and dispersity.

**Scheme 27:**
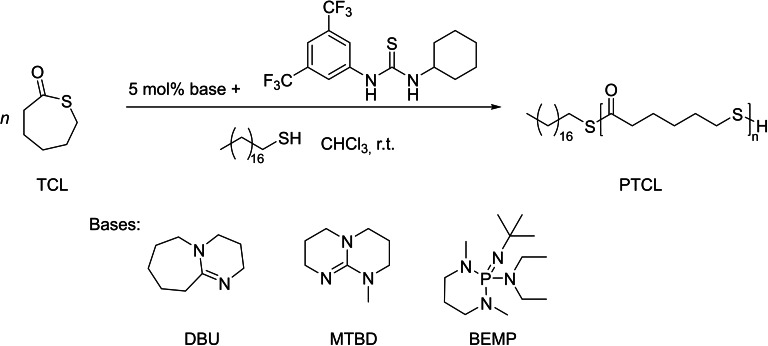
ROP of ϵ‐thiocaprolactone (TCL) promoted by a basic organocatalyst and eventual addition of a thiourea.^[36]^

**Scheme 28:**
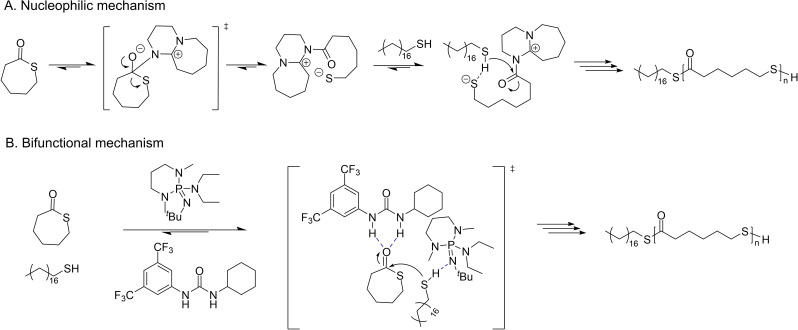
Proposed mechanisms for the ROP of TCL.^[36]^

In 2015, the group of Kiesewetter described the synthesis of well‐controlled PTCL by ROP of TCL using organocatalysts (Scheme 27).^[36]^ In the absence of a H‐bond donor, a series of bases such as tris[2‐(dimethylamino)ethyl]‐amine (Me_6_TREN), BEMP, DMAP, MTBD and DBU, were screened in CHCl_3_ at ambient temperature. Among them, MTBD and DBU were the most effective ones, promoting ROP in a controlled manner. This was confirmed by the linear growth of the experimental molar mass (*M*_n,SEC_ up to 32,000 g.mol^−1^) as a function of monomer conversion, while dispersities remained relatively narrow (*Đ*_M_=1.40–1.67); yet, these values broadened with increased reaction time, an indirect evidence of easy and fast transthioesterification reactions. The high activity of DBU and MTBD coupled with the observation that, on the other hand, the considerably basic but non‐nucleophilic BEMP is inoperative, revealed that the ROP of TCL likely follows a nucleophilic mechanism (Scheme 28): DBU and MTBD are not acting as general bases but rather promote ROP via nucleophilic attack at the thioester moiety.

A two‐component organocatalytic system featuring a H‐bond donor and a H‐bond acceptor moieties, has been demonstrated as a powerful strategy for the controlled ROP of cyclic esters.^[37]^ Thus, thiourea (a H‐bond donor) was investigated in the ROP of TCL. An NMR titration of TCL and thiourea revealed minimal activation of the monomer by thiourea, with a binding constant (*K*_eq_=2.7) much smaller than that of the parent oxo‐CL (*K*_eq_=42). DFT calculations further supported these NMR observations. Despite the small binding constant between TCL and thiourea, the H‐bond donor exhibits a marked effect on the ROP. The polymerization rate drastically increased upon addition of one equivalent of thiourea; on the other hand, the dispersity became somehow narrower (from *Đ*_M_=1.67 to 1.47), suggesting that monomer activation by thiourea may be operative despite the low binding constant. The linear growth of the experimental molar mass (*M*_n,SEC_) as a function of monomer conversion suggested a controlled character. When initiated in the presence of pyrenebutanol, the ROP of TCL catalyzed by this MTBD/thiourea combination featured similar ring‐opening kinetics, as when initiated from octadecanethiol; the resulting polymers exhibited overlapping RI and UV SEC traces, consistent with high end‐group fidelity. NMR studies were consistent with a chain‐end activation mechanism where the base activates the thiol proton by nucleophilic attack (Scheme 28); this contrasts with the traditional ROP organocatalysis towards polyesters wherein the chain‐end is activated through strong H‐bonding.^[38]^

On the other hand, high molar mass PTCL (*M*_n_=30,000 g.mol^−1^) was also prepared by lipase‐catalyzed polymerization of 6‐mercaptohexanoic acid.^[39]^ As expected, it was found that the *T*_m_ of the resulting PTCL (*ca*. 102 °C) was much higher than that of the corresponding oxo analogue PCL (*T*_m_=*ca*. 60 °C). More interestingly, the obtained PTCL was observed to degrade nearly quantitatively by the same lipase in toluene at 100 °C after 8 days to produce cyclic oligomers, mainly consisting of TCL and the dimer of TCL, which could reproduce the PTCL with nearly the same molar mass as the initial PTCL, providing another opportunity to develop recyclable plastics.

**Scheme 29:**
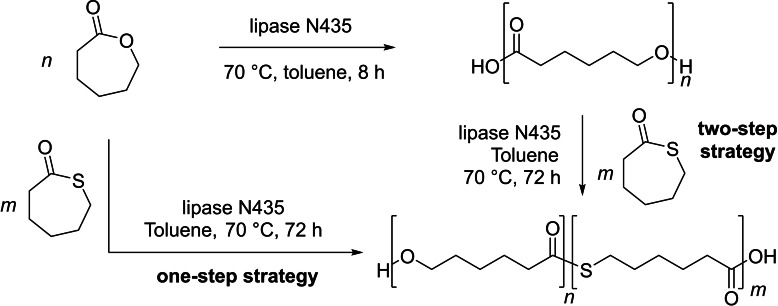
One‐ and two‐step strategies for copolymerization of ϵ‐thiocaprolactone and ϵ‐caprolactone.^[40]^

In 2017, the group of Avérous reported the enzymatic ROP of ϵ‐thiocaprolactone mediated by Novozyme 435, yielding the corresponding PTEs with low molar mass (*M*_n_=4,400 g.mol^−1^) and narrow dispersity (*Đ*_M_=1.10) (Scheme 29).^[40]^ The methodology was extended to the ROCOP of ϵ‐thiocaprolactone and ϵ‐caprolactone by the same lipase in toluene at 70 °C in one‐step or two‐step copolymerization strategies, giving rise to relatively low molar mass copolymers (*M*_n_=2900–10,800 g.mol^−1^). The copolymers resulting from the two strategies were apparently statistical in both cases (as determined by NMR and MALDI‐ToF MS); they featured *T*_m_ values in the range of 31–56 °C that are lower than those of the corresponding homopolymers (*T*_m_=61 °C for poly(ϵ‐caprolactone) and *T*_m_=55 °C for PTCL, respectively).

**Scheme 30:**
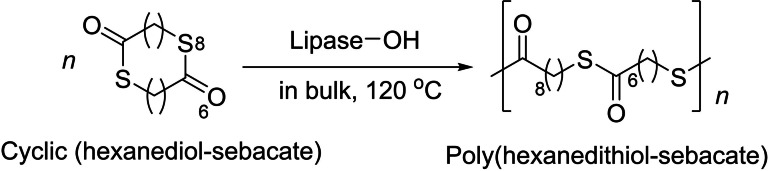
Lipase‐catalyzed ROP of a large cyclic thioester.^[41]^

In 2007, the group of Matsumura reported the enzymatic ROP of a cyclic hexanedithiol‐sebacate thioester mediated by a lipase (Scheme 30).^[41]^ The cyclic (hexanedithiol‐sebacate) monomer was synthesized from hexane‐1,6‐dithiol and dimethyl sebacate in the presence of immobilized lipase CA, yet in a low isolated yield (16%), as customary of macrocyclic monomers. The ROP reaction produced PTEs with high molar mass (*M*_w_=120,000 g.mol^−1^), significantly higher than that of the PTE obtained by direct polycondensation of the dithiol and diacid. The molar mass of the PTEs was influenced by the reaction conditions, such as reaction temperature, enzyme concentration, and reaction time. The resulting poly(hexanedithiol‐sebacate) had a higher melting temperature (*T*_m_=108.8 °C) than the corresponding ester analogue (*T*_m_=74.8 °C).

## 7 Ring‐opening polymerization of *S*‐carboxyanhydrides

**Scheme 31:**
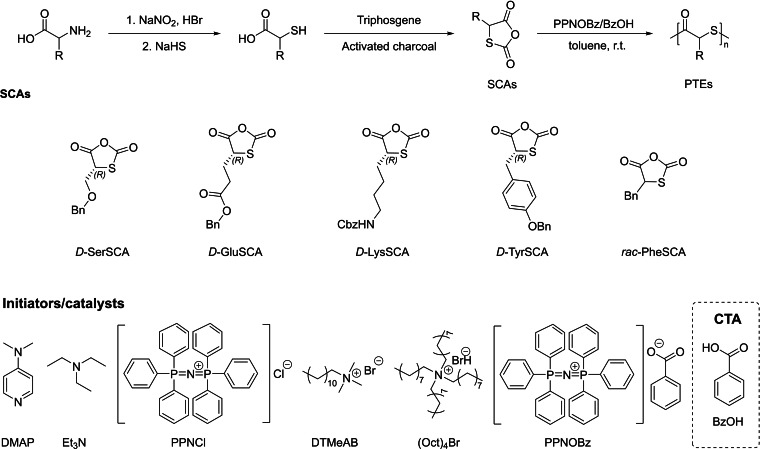
Typical synthesis of *S*‐carboxyanhydrides and their subsequent ROP using organocatalysts and a chain‐transfer agent (CTA).^[42]^

In 2021, the group of Tao reported the formation of PTEs from *S*‐carboxyanhydrides (SCAs) which can be synthesized from α‐amino acids in good yields (Scheme 31).^[42]^ A series of catalysts/initiators, such as TEA, DMAP, dodecyltrimethylammonium bromide (DTMeAB), tetraoctylammonium bromide [(Oct)_4_Br], and [PPN]Cl, were used to promote the ROP of *D*‐SerSCA. Among these, TEA‐ and DMAP‐based systems gave oligomers with molar mass lower than the theoretical values; the DTMeAB, (Oct)_4_Br, and [PPN]Cl systems mediated a poorly controlled ROP, as the observed molar mass values were significantly higher than the predicted ones. Surprisingly, the introduction of an acidic chain transfer agent (CTA) markedly favored the control of the molar mass of the resulting PTE, delivering ultrafast polymerization (TOF=*ca*. 1,500 min^−1^) and PTEs with narrow dispersity. The controlled nature of these ROPs was confirmed by the quite good agreement between the experimental molar masses and the theoretical ones calculated from the conversion and [SCA]_0_/[CTA]_0_ ratios, the linear growth of the experimental molar masses as a function of monomer conversion coupled with narrow dispersities (*Đ*_M_<1.3), and the chain extension experiment (*i. e*., upon addition of extra monomer).

Most importantly, the stereoregularity of the isolated PTE prepared with the [PPN]OBz/BzOH system was retained, indicating that this system does not promote racemization of SCAs nor of the resulting PTEs.

A remarkable feature of this approach is its versatile functionality due to the diverse structures of starting α‐amino acids. Hence, this chemistry was successfully extended to the ROP of *D*‐GluSCA, *D*‐LysSCA, *D*‐TyrSCA, and *rac*‐PheSCA (Scheme 31). All polymerizations were rapid and controlled, similarly to the ROP of *D*‐SerSCA. A random or sequential copolymerization of *D*‐SerSCA and *D*‐GluSCA gave a well‐defined random or block copolymer, respectively. Valuably, the ROP reaction is water‐insensitive and proceeds smoothly under air. Overall, these ROPs of SCA monomers offer control and tuning of molar mass, dispersity, stereoregularity, and side‐chain functionalities. Similarly to polydithiolactones,^[33]^ preliminary studies showed that these isolated PTEs prepared from SCAs feature good depolymerization ability under mild conditions (triethylamine as catalyst in CDCl_3_ at room temperature), giving a quantitative recovery of the corresponding cyclic dithioester monomers.

**Scheme 32:**
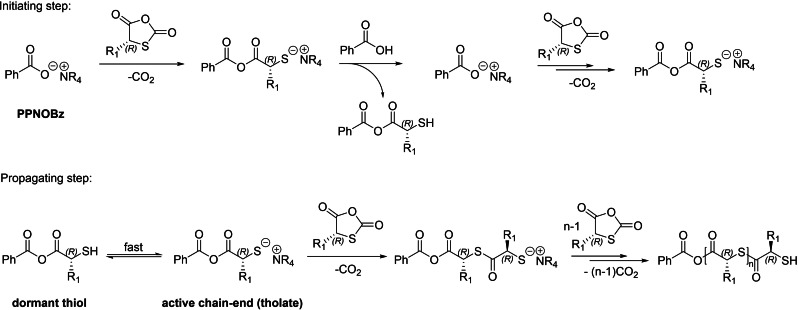
Plausible mechanistic pathway for the [PPN]OBz/BzOH‐mediated ROP of SCAs based on DFT calculations.^[42]^

DFT computations supported that the selectivity towards monomer propagation over transthioesterification is a consequence of the high polymerizability of SCAs. Also, the calculated energy barrier for chain back‐biting involving nucleophilic attack onto the terminal anhydride is higher than that of chain propagation. Based on these results, a plausible mechanism for the ROP of SCAs was proposed, as modelled with [PPN]OBz (the best initiator established experimentally) (Scheme 32). The reaction starts with the nucleophilic attack of [PPN]OBz onto the highly reactive monomer, with concomitant release of CO_2_. Then, the formed thiolate is protonated by the carboxylic acid used as CTA, generating a thiol and another carboxylate. This initiation step proceeds iteratively until all carboxylic acid is consumed. After that, proton transfer between dormant (thiol) and active chain‐ends (thiolate) enables propagation. Owing to the large acidity discrepancy between the initiating and propagating species, chain growth shows an induction period, as evidenced by experimental kinetic studies.

## 8 Isomerizing ring‐opening polymerization of thionolactones

Thionolactones are another category of sulfur‐containing monomer for synthesizing PTEs. The advantage of these monomers is that they can be, as a comonomer, incorporated into polyacrylates or polyolefins by radical ring‐opening polymerization, thereby combining the advantages of both radical polymerization and ROP, to produce biodegradable polymers for biomedical applications.^[43]^ Those reactions are yet out of the scope of the present review and, although thionolactones have attracted much attention over the past few years in polymer science, only salient homopolymers obtained from the ROP of thionolactones are discussed herein.

**Scheme 33:**
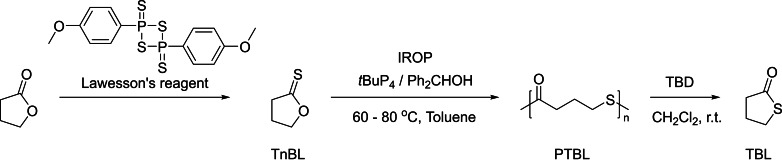
Synthesis and isomerizing ROP of γ‐thionolactones.^[44]^

A recent contribution by the group of Hong reports the formation of sustainable and degradable poly(γ‐thionobutyrolactone)s (PγTBLs) by polymerization of challenging five‐membered γ‐thionolactones (Scheme 33). The reaction is thermodynamically driven by the S/O isomerization instead of ring‐strain relief, and thus referred to as isomerization ROP (IROP).^[44]^ The controlled IROP of TnBL takes place in the presence of the strongly basic phosphazene ^*t*^BuP_4_ and Ph_2_CHOH; it was typically performed in highly concentrated toluene solution, the high monomer concentration being a critical factor for driving the IROP, at 80 °C within a few hours, forming PTBLs with high selectivity (up to 97%). The controlled nature of this IROP was confirmed by the linear growth of the experimental molar mass as a function of monomer feed ratio (at full monomer conversion), along with relatively narrow dispersity (*Đ*_M_ <1.6). *M*_n,SEC_ as high as 162,600 g.mol^−1^ could be reached, while the previously reported cationic ROP of γ‐thionobutyrolactone generated oligomers with molar mass values that did not exceed 6300 g.mol^−1^ along with broad dispersity (*Đ*_M_ >2.2).^[45]^

The high molar mass PTBL obtained revealed a semicrystalline thermoplastic with high melting and degradation temperatures (*T*_m_=100 °C; *T*_d_^5%^=230 °C), which exhibits good mechanical and hydrophilic performance (*M*_n_=162.6 kg mol^−1^: Young's modulus, *E*=296.5 MPa; yield strength, σ_y_=15.7 MPa; tensile strength, σ_b_=29.8 MPa; elongation at break, ε_B_=412.5%; and static contact angle, *θ*=78.4°). This kind of PγTE features thermal and mechanical properties that are competitive with commercial low‐density polyethylene (aka LDPE: *T*_m_=*ca*. 105 °C, *E*=*ca*. 280 MPa, σ_y_=*ca*. 8 MPa, σ_b_=*ca*. 10 MPa, ε_B_=*ca*. 200%, *θ*=*ca*. 102°).^[44]^ This suggests the utility of PTBL as an alternative to petroleum‐based non‐degradable plastics. In fact, the organocatalyst TBD efficiently promotes the degradation of PTBL, quantitatively converting it into its γ‐thiobutyrolactone, bestowing a valuable end‐of‐life to such polymers.

**Scheme 34:**
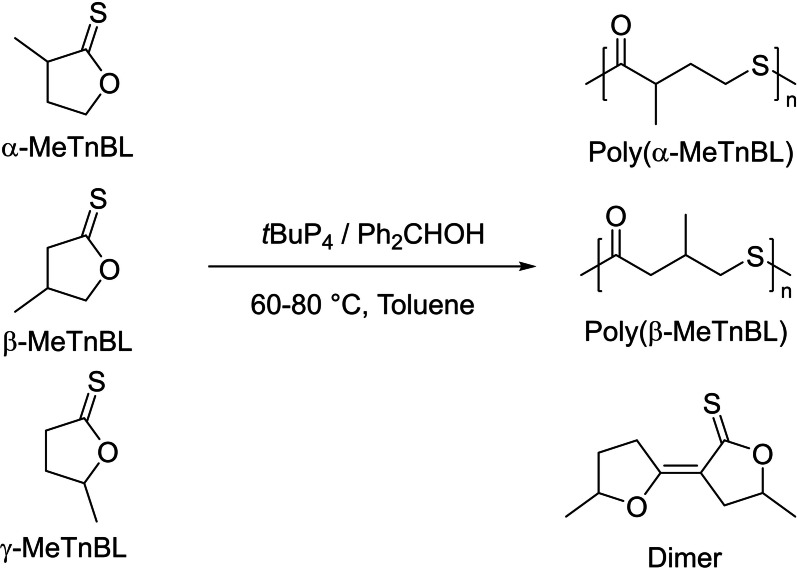
Generalization of the IROP strategy to related γ‐thionolactones.^[44]^

The generality of the above IROP strategy was extended to methyl‐substituted TnBL derivatives (Scheme 34); this included α‐methyl‐γ‐thionobutyrolactone (α‐MeTnBL), β‐methyl‐γ‐thionobutyrolactone (β‐MeTnBL) and γ‐methyl‐γ‐thionobutyrolactone (γ‐MeTnBL), which can be prepared via one‐step sulfurization of the corresponding methyl‐substituted γ‐butyrolactones.^[44]^ Under the optimized conditions established for the IROP of TnBL, rapid and selective polymerizations of α‐MeTnBL and β‐MeTnBL were also achieved by the combination of *t*BuP_4_ and Ph_2_CHOH, providing the corresponding PγTEs with high molar mass. However, when switching to γ‐MeTnBL, the use of the above catalytic system only formed the dimer of γ‐MeTnBL.

**Scheme 35:**
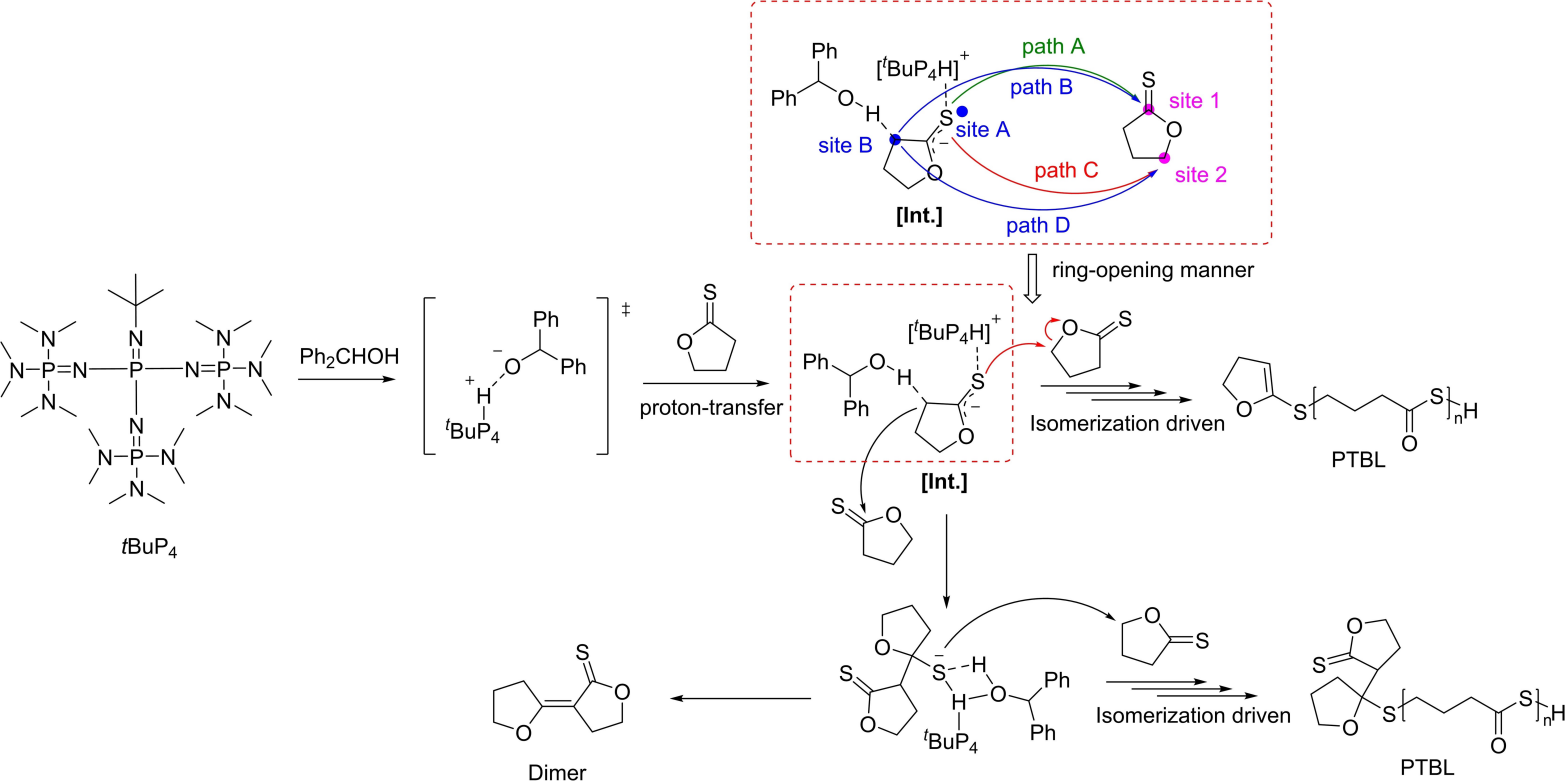
Possible mechanistic pathway for the isomerizing ROP of TnBL.^[44]^

Further mechanistic insights were gained from DFT computational studies (Scheme 35).^[44]^ The calculated energy profile showed that the energy barrier of the proton‐transfer pathway facilitated by Ph_2_CHOH‐mediated deprotonation is substantially lower than that of the nucleophilic pathway. This clearly suggests that the polymerization is selectively initiated through the kinetically favorable proton transfer pathway; this is supported by the observation of the HS‐ end‐group of low molar mass PTBL analyzed by ESI MS. Noteworthy, not only does Ph_2_CHOH play an important role in facilitating the deprotonation process, it also effectively suppresses backbiting side reactions during propagation by acting as an H‐bonding donor to stabilize the −C(O)S^−^ propagation species along with [*t*BuP_4_H]^+^, thereby accounting for the controlled IROP of such TnBLs. Four different TBL ring‐opening modes, namely paths A, B, C and D, were considered (Scheme 35): the formed enolate anion active species ([Int.]) could attack another TBL monomer via two reactive sites, site 1 and site 2, by either enolate‐S^−^ (site A) or enolate‐C^−^ (site B). The energy profile of these four ring‐opening modes (propagating step), and in particular the much lower energy barrier of path C, revealed the origin of the thermodynamically advantageous S/O isomerization product: the exclusive cleavage of the O‐alkyl bond of TnBL (path C) rather than of the more commonly opened O‐acyl bond (paths A and B) provides an overall thermodynamic driving force for the ROP of non‐strained γ‐thionobutyrolactones at room temperature or above towards PTE.

## 9 Concluding remarks and outcome

This short review highlights the significant progress made over the past half‐decade on the synthesis of polythioesters by ROP of thiolactones, *S*‐carboxyanhydrides or thionolactones, and ROCOP of thioanhydrides or thiolactones with epoxides or episulfides. Thiolactones have long remained challenging for polymer chemists due to their high reactivity and poor control over their polymerization, as compared to those achieved with their corresponding cyclic oxoesters. Yet, better control can be expected in due course by further optimization of the initiator structure and polymerization process. In particular, further improvements may be anticipated for the stereocontrolled ROP of chiral thiolactones using well‐defined complexes. Although the formation of polythioesters from homopolymerization of thionolactones are rare, it opens up another new opportunity to introduce sulfur atoms into the backbone of aliphatic polyesters by ROCOP of cyclic esters, especially poly(ϵ‐caprolactone), due to its low melting temperature that limits its practical applications. Many of the polythioesters reported in recent years have shown great potential to their recyclability. It is reasonable to believe that the introduction of fused *trans*‐rings, such as cyclohexyl and phenylene, into ϵ‐thiocaprolactone, may make a “monomer‐polymer‐monomer” loop possible. Also, it can be anticipated that environmentally friendly PTEs will be considered in the future as useful complements to polyesters and alternatives to replace conventional fossil‐based plastics, especially thanks to their facile degradation and high potential for recyclability.

## Conflict of interest

The authors declare no conflict of interest.

## Biographical Information

*Hui Li studied at the University of Chinese Academy of Sciences and got his Master of Engineering in 2018, where he worked on organocatalytic ring‐opening polymerization of lactones. For his on‐going doctoral studies, he joined the group of Prof. Jean‐François Carpentier and Dr. Sophie M. Guillaume at the University of Rennes 1. His current research focuses on organic/organometallic catalytic systems for stereoselective ring‐opening polymerization of original functional β‐lactones and cyclic thioesters*.

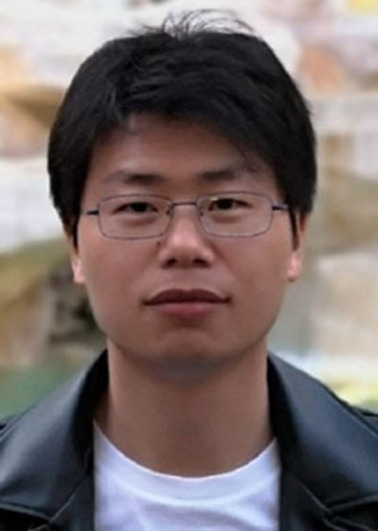

## Biographical Information

*Sophie M. Guillaume received her PhD in Inorganic/Organometallic Chemistry from the University of Syracuse, New York, USA, and afterwards joined the CEA, Saclay, France, for her postdoctoral research. She then joined the CNRS and moved to the LCPO, Bordeaux, France. She now holds a CNRS Directeur de Recherche position at the University of Rennes, France. Her research is mainly focused on polymer chemistry and polymerization catalysis, on the synthesis and structure‐property relationships of synthetic polymers (especially polyesters, polycarbonates, polyolefins, polyurethanes). Areas of emphasis include bio‐based degradable polymers and functionalized and reactive (co)polymers for advanced industrial and biomedical applications. Besides, since 2022, she acts as deputy director of the Rennes Institute of Chemical Science (ISCR‐Rennes)* .

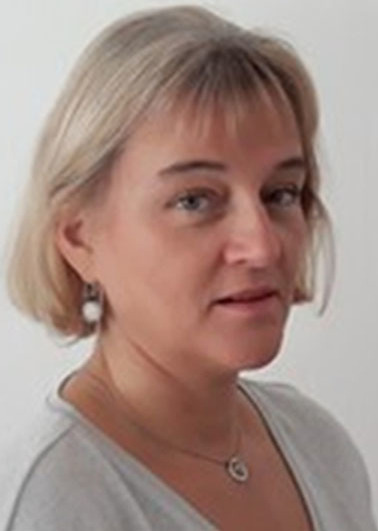

## Biographical Information

*Jean‐François Carpentier received his PhD in molecular catalysis from the University of Lille in 1992 with A. Mortreux and was a Postdoctoral Associate at U. Iowa with R. F. Jordan. After a CNRS research fellow position, dedicated to late transition metal catalysis for C−C and C−H bond formation, he moved in 2001 to the University of Rennes as full Professor. His current research interests lie in the organometallic chemistry of oxophilic elements and their use in catalysis for polymer engineering and fine chemicals synthesis. In 2005, he was elected member of the Institut Universitaire de France. In 2014, he was awarded the Silver CNRS medal and the prix Lequeux from the French Academy of Sciences. Besides, since 2016, he acts as vice‐president in charge of research of the University of Rennes 1*.

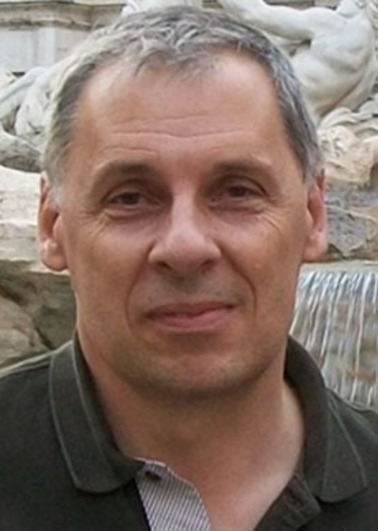

## Footnotes

n1 In the ROP of chiral cyclic monomers, the degree of stereoregularity is commonly expressed as the probability of *racemic* or *meso* enchainment (probability of generating a next *racemic* (syndiotactic) or *meso* (isotactic) diad), *P*_r_ and *P*_m_ (*P*_m_=1–*P*_r_), respectively. *P*_r_=*P*_m_=0.50 describes a completely atactic polymer; *P*_m_=1.00 (i. e., *P*_r_=0.00) and *P*_m_= 0.00 (i. e., *P*_r_=1.00) describe perfectly isotactic and syndiotactic polymers, respectively. These parameters can be calculated from the deconvoluted homonuclear decoupled ^1^H NMR and/or quantitative ^13^C NMR spectra.
